# Inhibiting the RNA helicase DDX3X in Burkitt lymphoma induces oxydative stress and impedes tumor progression in xenografts

**DOI:** 10.3389/fcell.2025.1642006

**Published:** 2025-07-23

**Authors:** Hugues Beauchemin, Zeinab Dalloul, Eva-Maria Piskor, Virginie Calderon, Andrew Chatr-aryamontri, Thierry Bertomeu, Tarik Möröy

**Affiliations:** ^1^ Institut de Recherches Cliniques de Montréal (IRCM), Université de Montréal, Montréal, QC, Canada; ^2^ Department of Medicine, Division of Clinical and Translational Research, McGill University, Montreal, QC, Canada; ^3^ The ChemoGenix Platform, Institut de Recherche en Immunologie et Cancer (IRIC), Université de Montréal, Montreal, QC, Canada; ^4^ Département de Microbiologie, Infectiologie et Immunologie, Faculty of Medicine, Université de Montréal, Montreal, QC, Canada

**Keywords:** RNA helicase, DDX3 as a potential target, Burkitt lymphoma (BL), DDX3 inhibitor, xenograft, ATP-dependent RNA helicase

## Abstract

**Introduction:**

Burkitt Lymphoma (BL), an aggressive B-cell lymphoma driven by MYC translocations, requires intensive chemotherapy treatments which deliver high effectiveness yet increase future risks of developing secondary malignancies. We have previously shown that DDX3X, an RNA helicase frequently mutated in BL, is essential for B cell lymphomagenesis in mice.

**Methods and results:**

To assess if DDX3X could therefore represent a promising therapeutic target for BL, we tested two DDX3X inhibitors, the well characterized RK-33 and the more potent newly developed C1, in three BL cell lines (CA46, Raji, Daudi). We found that the 3 cell lines exhibited differential sensitivities to the drugs *in vitro,* with Daudi being the most susceptible and Raji the most resistant. *In vivo,* RK-33 treatment in a xenograft BL model reduced tumor progression in all cell lines, albeit with variable efficacy compared to the clinical drug Pevonedistat, and again with the Daudi cells being the most responsive to the treatment. Transcriptomic and proteomic analyses indicated that RK-33-mediated inhibition of DDX3X, and DDX3X ablation through siRNA affects oxidative phosphorylation among other pathways and leads to an increase of intracellular reactive oxygen species (ROS). A CRISPR chemogenomic screen to identify synthetic lethalities linked to RK-33 implicated enzymes of the glutathione synthesis pathway and the Keap1-Nrf2-ARE pathway. We therefore tested the inhibition of the glutathione pathway with buthionine sulfoximine and showed that it reduced the CC50 of RK-33 in BL cells lines.

**Conclusion:**

Our findings not only support DDX3X as a therapeutic target in BL but also provide evidence for a combinatorial treatment strategy to improve the efficacy of current treatments.

## Introduction

Burkitt Lymphoma (BL) is a highly aggressive blood cancer derived from germinal center B cells (Molyneux et al., 2012; Bouska et al., 2017; Miard et al., 2017). This malignancy is driven by the *c-MYC* oncogene, which is transcriptionally activated by chromosomal translocations to enhancers and promotes of the immunoglobulin (Ig) µ heavy- or the λ light chain loci (Gostissa et al., 2009). Treatment options for BL include standard chemotherapeutic agents (cyclophosphamide, vincristine, methotrexate, doxorubicin, and cytarabine), although some targeted therapies have shown success (e.g., Rituximab) (Bouska et al., 2017; Short et al., 2017; Egan et al., 2019). When caught early (stage I or II), long-term survival rates in children are 90% or greater. However, in later stages (III and IV) and in older children, the survival rates drop to 80%–90% (Short et al., 2017). Of great concern are reports of secondary cancers arising later in life in previously treated pediatric BL patients, most likely due to DNA damage caused by the chemotherapeutic agents used (Abrahão et al., 2020; Zahnreich and Schmidberger, 2021). There is, therefore, a great need to identify new therapeutic approaches that would reduce the toxicity in BL therapies.

DDX3X is an RNA-helicase that unwinds RNA-RNA and RNA-DNA strands, remodels RNA-protein complexes and regulates mRNA translation (Song and Ji, 2019; Mo et al., 2021; Lacroix et al., 2023), but has recently also be identified as a critical factor in the trafficking of cell surface receptors such as PD-L1 (Chen et al., 2024). The DDX3X protein is highly conserved between mice and humans (98%) and contains a helicase and an ATP-binding domain (Mo et al., 2021). *DDX3X* is located on the X chromosome and escapes X-chromosome inactivation in humans and mice (Berletch et al., 2011). Consequently, females have two active *DDX3X* copies, while males carry only one, although the Y chromosome harbors a homolog called *DDX3Y* that encodes a nearly identical protein (>90% homology) (Ditton et al., 2004). The DDX3Y protein is absent from human B cells (Ramathal et al., 2015) but is expressed in murine spleen (Lacroix et al., 2022a). Importantly, somatic loss of function (LOF) mutations that inactivate the helicase domain on one allele of the *DDX3X* gene are frequent in BL (>30% of cases) (Richter et al., 2012; Bouska et al., 2017; Kaymaz et al., 2017; Moffitt and Dave, 2017; Grande et al., 2019; Lopez et al., 2019) and occur preferentially (>90%) in males. It has been reported that male BL cells with DDX3X LOF mutations upregulate DDX3Y (Gong et al., 2021), probably because DDX3 activity is required for RNA-dependent cellular processes. It is thus likely that DDX3Y compensates the loss of DDX3X and suggests that DDX3 proteins are required for malignant transformation and are not tumor suppressors.

Recent studies have demonstrated that male human BL cell lines and male primary BL with somatic LOF *DDX3X* mutations upregulate expression of the DDX3Y protein, probably through post-transcriptional regulation of mRNA stability (Gong et al., 2021; Rengarajan et al., 2025). In female BL somatic *DDX3X* LOF mutations are very rare and occur in only one allele, leaving the other allele intact (Richter et al., 2012; Schmitz et al., 2012; Lopez et al., 2019; Gong et al., 2021). In addition, our own studies with transgenic mice that are prone to develop B cell lymphomas owing to a MYC transgene that is under the control of the immunoglobulin heavy chain or -lambda light chain enhancer and therefore constitutively expressed in follicular B cells or pre-germinal center B cells, respectively, confirmed this finding (Lacroix et al., 2022a). B cell lymphomagenesis was significantly impeded, or even abrogated in female MYC transgenic mice when the *Ddx3x* gene was deleted (Lacroix et al., 2022a). In male mice *Ddx3x* deletion led to the upregulation of *Ddx3y* and rescued partially B cell lymphomagenesis (Lacroix et al., 2022a). This suggested that DDX3 activity in general is required for a c-*MYC*-driven BL and would therefore provide a novel therapeutic target (Lacroix et al., 2022a; Lacroix et al., 2022b).

RK-33 (diimidazo [4,5-d:4′,5′-f]-[1,3] diazepine) is a first-in-class small molecule inhibitor of DDX3 that causes G1 cell cycle arrest, apoptosis, and radiation sensitization (Bol et al., 2015a; Xie et al., 2016; Heerma van Voss et al., 2018a; Tantravedi et al., 2019). RK-33 binds specifically to DDX3X and -Y within their ATP binding regions at low μM concentrations, but not to the closely related proteins DDX5 and DDX17 (Xie et al., 2016). RK-33 is not toxic in mice up to 20 mg/kg and accumulates at therapeutic doses in various organs supporting its relevance as a chemotherapeutic drug, although it has not yet entered phase I clinical trials in human, as research to date has been confined to preclinical studies (Bol et al., 2015a; Bol et al., 2015b). RK-33 targets the ATPase binding pocket and inhibits the helicase activity of DDX3 activity. It was tested in animal models for several human cancers including chronic myeloid leukemia, lung cancer, breast cancer, prostate cancer, and medulloblastoma (Bol et al., 2015a; Xie et al., 2016; Tantravedi et al., 2019; Winnard et al., 2024; Duan et al., 2025). Other DDX3 inhibitors have recently been identified using high-throughput RNA helicase assays (Nakao et al., 2020) for the Eukaryotic Translation Initiation Factor 4A3 (eIF4A3), which is like DDX3 a DEAD box ATP-dependent RNA-helicase (Asthana et al., 2022). Several eIF4A3 inhibitors showed a positive correlation between their ATPase inhibitory activity and helicase inhibitory activity (Nakao et al., 2020). Since the ATP-binding sites of eIF4A3 and DDX3X are very similar, further screening allowed to identify a new DDX3X helicase inhibitor, called C1 (Nakao et al., 2020). This molecule has a stronger helicase inhibitory activity for DDX3X than for eIF4A3 (Nakao et al., 2020).

Here, we have tested the effect of the DDX3 inhibitors RK-33 or C1 on three BL lines: CA46, which carries a wt *DDX3X* allele, but has lost its Y chromosome and therefore cannot upregulate the male DDX3X paralogue DDX3Y; Raji, which has a *DDX3X* ^320-342^ LOF deletion, but expresses *DDX3Y* (Gong et al., 2021); and Daudi, in which DDX3X carries a single amino acid variations outside its helicase domain but still relies on DDX3Y since DDX3X expression is below detection levels. We describe the sensitivity of all cell lines to RK-33 and C1 and show their effect *in vivo* on BL xenografts compared to the clinical drug Pevonedistat. Transcriptomic and proteomic analyses as well as chemogenomic screens indicated that RK-33 and DDX3X knockdown affect oxidative phosphorylation and generate reactive oxygen species (ROS). We identified the inhibition of the Glutathione synthesis pathway to be synthetically lethal with the inhibition of DDX3X by RK-33 in BL cells, suggesting novel strategies for combinatorial therapies.

## Materials and methods

### Cell lines, inhibitors and western blot antibodies

The Raji, DG-75 and Ramos Burkitt lymphoma (BL) cell lines were authenticated using ATCC’s STR Profiling Authentication Services. The CA46, Daudi, GA-10, EB1 and ST486 cell lines were obtained from ATCC, while the BL41 cell line was obtained from DMSZ. The Namalwa cell line was a generous gift from Dr. Javier Di Noia at IRCM (Montreal, QC, Canada). Daudi-Fluc-Puro and Raji-Fluc-Puro cell lines were obtained from Imanis Life Sciences. Data concerning mutation in the gene DDX3X carried by the different cell lines was extracted from the Broad depmap portal (https://depmap.org/portal).

The RK-33 inhibitor was purchased from Focus Biomolecules and prepared as a 50 mM stock solution in dimethyl sulfoxide (DMSO) for both *in vitro* and *in vivo* experiments. MLN4924 (pevonedistat) was obtained from AdooQ BioScience and prepared as a 50 mM stock solution in DMSO for *in vivo* experiments. The C1 compound was custom synthesized by ChemSpace (Latvia) and prepared as a 50 mM stock solution in DMSO.

DL-buthionine-sulfoximine (BSO) and inosine were purchased from Millipore Sigma and prepared as 100 mM stock solutions in water. TAK-243 and omaveloxolone were obtained from Selleck Chemicals, while CPUY192018 was obtained from MedChemExpress Co., Ltd.; all were prepared as 100 mM stock solutions in DMSO.

For Western blot, The DDX3Y-specific antibody was purified from rabbits immunized as published (Gong et al., 2021) by Biomatik. The DDX3X antibody A300-474A was from Bethyl laboratory.

### Mice

NSG (NOD.Cg-*Prkdc^scid^ Il2^rgtm1Wjl^*/SzJ, Strain #:005557, RRID:IMSR_JAX:005557) mice were obtained from Jackson Laboratory and bred in the IRCM SPF+ (free of pathogens and opportunistic germs) animal facility. Mice were maintained on a 12-h light/dark cycle with *ad libitum* access to food and water.

All animal experiments were reviewed and approved by the Animal Care Committee of IRCM (Protocol #2025–1282) in accordance with the guidelines of the Canadian Council on Animal Care.

### Cell viability and apoptosis assay

Cell viability for all cell lines was determined using an XTT assay from Biotium following the manufacturer’s instructions. Briefly, 5 × 10^3^ to 1 × 10^4^ cells were seeded in 96-well plates with 100 μL of RPMI supplemented with 10% FBS, containing serial dilutions of the compounds from 100 μM down to 1% DMSO (vehicle control). Cells were cultured at 37°C for 4 days. 50 μL of XTT labeling reagent was then added to each well, and plates were incubated for 1 h at 37°C. Absorbance was measured at 490 nm (signal) and 650 nm (background) using a SpectraMax 190 Microplate Reader (Molecular Devices) with SoftMax Pro 5.2 software. Cell viability was calculated by subtracting 650 nm background values from 490 nm absorbance values and normalizing to the DMSO-treated control. Nonlinear regression curves and the 50% cytotoxic concentration (CC_50_) were determined using GraphPad Prism version 10.4.2.

The synthetic lethality and/or rescue potential of combinational treatments was also assessed using the XTT assay. The CC_50_ values for additional drugs were determined as described above and were then used at concentrations below their CC_50_: DL-buthionine-sulfoximine (10 μM); inosine (500 μM); TAK-243 (4 nM); omaveloxolone (50 nM); CPUY192018 (5 μM). These compounds were combined with serial dilutions of RK-33 (ranging from 150 μM to 1% DMSO vehicle control) to evaluate combinatorial effects.

To assess apoptosis induced by DDX3X inhibitors, Raji, CA46, and Daudi cells were treated for 24 h with RK-33 at concentrations ranging from 0 to 50 μM and C1 at 0–5 μM. Cells were then stained with FITC-labeled annexin V (BioLegend) and propidium iodide (PI), acquired using a Sony SA3800 Spectral Cell Analyzer, and analyzed with FlowJo (version 10.9.0).

### Xenografts

To establish a luciferase-expressing CA46 cell line, parental CA46 cells were transfected with the pF CAG luc hygro plasmid (a gift from Brett Stringer; Addgene plasmid #67502; http://n2t.net/addgene:67502; RRID: Addgene_67502). This plasmid contains the firefly luciferase gene under the control of the chicken beta-actin promoter and hygromycin resistance gene (Stringer et al., 2019). Selection was carried out using hygromycin B (Millipore Sigma) in a methylcellulose-based semi-solid medium (ClonaCell-TCS, Stemcell Technologies). The resulting CA46-Fluc-Hygro cell line was compared to the commercially available Raji-Fluc-Puro and Daudi-Fluc-Puro cell lines, exhibiting a bioluminescence level approximately 1/30 of the latter two (data not shown).

To determine the optimal cell number for the lymphoma dissemination monitoring, five-week-old NSG mice were injected intravenously (i.v.) with increasing doses (1 × 10^5^, 2 × 10^5^, 3 × 10^5^, 4 × 10^5^, and 5 × 10^5^ cells in 100 µL PBS) of Fluc-expressing BL cell lines. Dissemination was evaluated using bioluminescence imaging with the IVIS Imaging System 200 (Xenogen). Based on these results, the following cell numbers were selected: Raji-Fluc-Puro: 1 × 10^5^; Daudi-Fluc-Puro: 2.5 × 10^5^; CA46-Fluc-Hygro: 5 × 10^5^.

For each cell line, 20 five-week-old NSG mice were injected i.v. with the optimized number of cells in 100 µL PBS and mice were randomized into control (vehicle-treated) and treatment (RK-33 or Pevonedistat) groups. For bioluminescence imaging, mice were injected intraperitoneally (i.p.) with 150 mg/kg D-luciferin (15 mg/mL in PBS; 250 µL (∼3.75 mg) per 25 g mouse) and anesthetized using 2% isoflurane. Imaging was performed with the Xenogen IVIS Imaging System 200 using exposure times of 1–30 s. A maximum of five mice were imaged simultaneously, with continuous isoflurane exposure to maintain anesthesia. Bioluminescence was recorded every other day, and mice reaching a predefined dissemination threshold, set at an average radiance of 1 × 10^6^ photons/s/cm^2^/sr for both Raji and Daudi xenografts, and 3 × 10^5^ photons/s/cm^2^/sr for the CA46 xenograft, were assigned to treatment. Mice in the RK-33 treatment group received 50 mg/kg RK-33 (5 mg/mL in 20% DMSO/corn oil, 250 µL per 25 g mouse, corresponding to 1.25 mg), administered i.p. every other day for 2 weeks. For the Pevonedistat treatment group, mice received subcutaneous injections of 90 mg/kg Pevonedistat (14.7 mg/mL in 5% DMSO/30% PEG 300/5% Tween 80, 150 µL per 25 g mouse, corresponding to 2.2 mg) every other day for 2 weeks. Control mice received vehicle injections consisting either of 10 µL of 20% DMSO in corn oil per gram of body weight or 150 µL of 5% DMSO/30% PEG 300/5% Tween 80, depending on the treatment group at the same frequency. Lymphoma progression was monitored every other day for 3 weeks after treatment initiation or until mice reached endpoint criteria. Results were reported as the radiance fold change over the first day of treatment. BL progression curves were compared by a CGGC permutation test using 10,000 permutations (Elso et al., 2004).

Due to differences in bioluminescence intensity among cell lines, sample imaging was standardized for visual comparison. Imaging of mice injected with Raji-Fluc-Puro or Daudi-Fluc-Puro cells was conducted with an exposure time of one second, while imaging of mice injected with CA46-Fluc-Hygro cells required an exposure time of 30 seconds due to the lower signal intensity.

### siRNA-mediated knockdown of DDX3X and DDX3Y

To target the human DDX3X and DDX3Y genes, Small interfering RNAs (siRNAs) were obtained from Origene. The siRNAs used were the Trilencer-27 siRNA oligo duplex kits SR319906BL for DDX3X, SR305689BL for DDX3Y, and the universal scrambled negative control siRNA duplex (SR30004). For the RNA-seq experiments, 2.5 × 10^5^ Raji and CA46 cells were electroporated with 10 pmol of either the scrambled control, DDX3X, DDX3Y, or a combination of DDX3X and DDX3Y siRNAs using the Neon Transfection System (Thermo Fisher Scientific) and the 10 μL Neon Transfection kit with Buffer T. The parameters were as follow: for Raji cells, a pulse voltage (PV) of 1,100 V, pulse width (PW) of 30 ms, and pulse number (PN) of 2; for CA46 cells, a PV of 1,150 V, PW of 20 ms, and PN of 2. For the mass spectrometry experiments, 2.5 × 10^6^ cells were electroporated with 100 pmol of siRNA using the 100 μL Neon kit with the same electroporation parameters. After the electroporation, cells were seeded into 24-well culture plates and incubated for 24 h in RPMI medium supplemented with 10% fetal bovine serum (FBS), then harvested for RNA or protein extraction. All these conditions were done in duplicate.

### Primary human B cell isolation and culture

This research involves human participants who gave informed consent to participate in the study under the approbation of the research ethics committees of Héma-Québec (Project # 2021–016) and received approval from the Research ethics committee (CÉR) of the IRCM (protocol # 2022–1138). Three healthy male donors provided primary human B cells which were derived from leukapheresis products. Héma-Québec supplied patient samples that were stored in Leukoreduction System (LRS) Chambers (Excellos). The leukocyte/red blood cell mixture collected from the chambers was separated by density gradient centrifugation using Lympholyte®-H (Cedarlane) to isolate peripheral blood mononuclear cells (PBMCs). Untouched B cells were then purified from the PBMC fraction using the MojoSort™ Human Pan B Cell Isolation Kit (BioLegend). Isolated B cells were resuspended in RPMI medium supplemented with 20% charcoal-stripped FBS, 10 mM HEPES and 55 nM β-Mercapthoethanol and a total of 1 × 10^6^ purified B cells were seeded into 12-well plates pre-coated with an irradiated feeder cell layer (YK6-CD40L-IL21), which were provided as a generous gift by Dr. Daniel Hodson (Wellcome-MRC Cambridge Stem Cell Institute, Cambridge, UK), and express CD40 ligand and secrete interleukin-21 to support B cell activation and proliferation (Caeser et al., 2019). Cells were cultured under these conditions for a 48 h activation period, and then treated with either 5 µM RK-33 or 0.4% DMSO (vehicle control) for 24 h. After treatment, B cells were carefully collected to prevent feeder cell contamination. Total RNA was then extracted for RNA sequencing.

### Transcriptome profiling

To assess the impact of DDX3X inhibition on the global transcriptome, total RNA was extracted from 2.5 × 10^5^ Raji and CA46 cells after 24-h treatments with either RK-33 (50 μM for Raji and 10 μM for CA46) or siRNAs targeting DDX3X, DDX3Y, or both DDX3X/DDX3Y and their respective controls (DMSO or Scramble siRNA). Similarly, total RNA was extracted from primary human B cells treated with RK-33 or DMSO for 24 h. In all cases, RNA was extracted using the RNeasy Mini Kit (Qiagen). RNA integrity and quality were verified using the RNA 6000 Pico Kit on an Agilent Bioanalyzer system.

RNA-seq libraries were prepared using the Illumina TruSeq Stranded mRNA Library Prep Kit, following the manufacturer’s protocol. Sequencing was conducted on an Illumina HiSeq 2000 system using the TruSeq PE Cluster Kit v3-cBot-HS. Quality control of the raw sequencing reads was performed with FASTQC v0.12.1. Reads were aligned to the human reference genome GRCh38 using STAR v2.7.11b, with an average of 87% of reads uniquely mapped. Raw read counts were obtained using FeatureCounts v2.0.6, based on the GRCh38 (release 110) genome annotation. Differential gene expression analysis was performed using the DESeq2 package in R. KEGG pathway enrichment analysis was performed using the GSEA (Gene Set Enrichment Analysis) tool with 1,000 gene set permutations (Joshi et al., 2013) or using ShinyGO v0.82, based on significantly deregulated genes (adjusted *P* < 0.05) (Ge et al., 2020). Expression heatmaps were created using the Heatmapper online tool, applying hierarchical clustering to both rows and columns using the Average Linkage method and Kendall’s Tau distance metric (Babicki et al., 2016). Principal component analysis (PCA) was done using the Statistics Kingdom platform (https://www.statskingdom.com). Additional functional enrichment analysis, including gene ontology and pathway analysis, was performed using the gprofiler2 package in R. All RNA-seq data are available through GEO under accession number GSE294862. A summary of RNA-seq data are available in Supplementary Tables S1–S3.

### Proteome profiling

To evaluate the impact of DDX3X inhibition on the chemogenomic interactions from nonglobal proteome, total protein was extracted from 2.5 × 10^6^ Raji and CA46 cells following 24-h treatments with RK-33, DMSO, or siRNAs targeting DDX3X, DDX3Y, or both. Cells were lysed in RIPA buffer supplemented with EDTA-free protease inhibitors (Roche) and disrupted by brief sonication (3 s) using a Branson 250 Digital Sonifier. Lysates were centrifuged, and supernatants were collected for downstream analysis.

Protein concentration was determined using the BCA Protein Assay Kit (Thermo Scientific). For each condition, approximately 100 µg of total protein was digested, isobarically labeled with TMT 6-plex reagents (Thermo Fisher Scientific) and fractionated into five parts. Mass spectrometry analysis was done on an Orbitrap Fusion mass spectrometer (Thermo Scientific). To control for labeling bias, samples were organized into four independent TMT 6-plex sets. Two sets (TMT-1 and TMT-2) each contained one replicate of CA46 cells (for a total of two biological replicates for each condition) treated with DMSO, RK-33, scramble siRNA, DDX3X siRNA, DDX3Y siRNA, and combined DDX3X/DDX3Y siRNAs. The remaining two sets (TMT-3 and TMT-4) included one replicate each of Raji cells similarly treated. Peptide identification and quantification were performed using Proteome Discoverer v2.1 (Thermo Scientific) against the UniProt human protein database (downloaded October 2024). Within each TMT experiment, relative protein abundances were normalized to total protein abundance per sample and corrected for TMT batch effects by first averaging the expression values for each gene across all samples within each TMT set. For each gene, a normalization ratio was then calculated between paired TMT sets (e.g., TMT-1/TMT-2 and TMT-3/TMT-4). This gene-specific ratio was used to scale the corresponding values in one TMT set (e.g., TMT-2), thereby minimizing inter-set variation and effectively correcting for batch effects. Proteins exhibiting a fold change ≥1.4 relative to their corresponding controls (DMSO for RK-33 treatment, scramble siRNA for siRNA-treated samples) were considered differentially expressed and selected for pathway enrichment analysis. Enrichment analysis of biological pathways was performed using GSEA and ShinyGO v0.82, following the same procedures applied to the transcriptomic dataset. Both upregulated and downregulated proteins were analyzed based on the 1.4-fold change threshold to identify significantly altered functional categories and pathways. Heatmaps and PCA were performed as described for the transcriptome profiling, using the same tools and parameters. Proteomic data are available in Supplementary Table S4.

### CRISPR/Cas9 screen for modulators of RK-33 sensitivity

To identify genetic alterations that either sensitize or confer resistance to DDX3X inhibition, a genome-wide pooled CRISPR/Cas9 knockout screen in the human pre-B acute lymphoblastic leukemia cell line NALM-6 was performed by the ChemoGenix platform at the Université de Montréal (Montreal, QC, Canada), as described previously (Bertomeu et al., 2018). Briefly, a NALM-6 clone stably expressing a doxycycline-inducible Cas9 made from pCW-Cas9 (Addgene #50661) was transduced with the EKO library, a genome-wide sgRNA targeting protein-coding genes (278,754 different sgRNAs). sgRNA-mediated gene disruption was then initiated by adding doxycycline (2 μg/mL) for a period of 7 days to allow for robust gene knockout across the population. Post-induction, the pooled library was then split into different T-75 flasks (28 × 10^6^ cells per flask; a representation of 100 cells/sgRNA) in 70 mL at 4 × 10^5^ cells/mL. One set was treated with 7 µM RK-33 and the other with DMSO as vehicle control. Cells were cultured under these conditions for 8 days, with monitoring of growth every 2 days, diluting back to 4 × 10^5^ cells/mL and adding more compound to maintain the same final concentration whenever cells reached 8 × 10^5^ cells/mL. Viable cells were harvested at the end of the treatment window.

Genomic DNA was isolated from cell pellets using the Gentra Puregene Cell Kit (Qiagen), following the manufacturer’s guidelines. The sgRNA cassette integrated into each genome was PCR-amplified as described (Bertomeu et al., 2018) and prepared for next-generation sequencing (NGS). Sequencing was performed using the Illumina NextSeq 2000 platform to determine sgRNA abundance in each treatment condition. Reads were aligned using Bowtie 2.4.4 in forward-strand only mode (using the norc parameter) with otherwise default parameters and total read counts per gRNA were tabulated. Context-dependent chemogenomic interaction scores were calculated using a modified version of the RANKS algorithm (Bertomeu et al., 2018) that uses guides targeting similarly essential genes as controls to distinguish condition-specific chemogenomic interactions from non-specific fitness/essentiality phenotypes. The chemogenomic screen data are available in Supplementary Table S5.

### Total reactive oxygen species measurement

Intracellular total reactive oxygen species (tROS) levels were measured using the Total ROS Assay Kit 520 nm from Thermo Fisher Scientific. Raji cells were treated with 12.5 μM RK-33 or DMSO, and CA46 cells were treated with 10 μM RK-33 or DMSO for 24 h prior to analysis. Cells were analyzed on a BD LSRFortessa™ Cell Analyzer (BD Biosciences) using the 488 nm excitation laser and FITC emission filter (530/30 nm). The extent of ROS production was quantified by comparing the mean fluorescence intensity (MFI) between RK-33-treated and vehicle-treated control cells.

## Results

### DDX3X inhibitors induce apoptosis in human Burkitt lymphoma cells

The sensitivity of Burkitt lymphoma (BL) cell lines to DDX3X inhibition was evaluated by treating BL cell lines with two different DDX3X inhibitors: RK-33 (Bol et al., 2015a; Xie et al., 2016; Yang et al., 2020) and the recently developed, more potent inhibitor C1 (Abate et al., 2015; Nakao et al., 2020). Nine BL cell lines were analyzed, six derived from male patients and three from female patients. Of the six male lines, two carried a wild-type (wt) DDX3X allele (CA46, BL41), while four harbored a mutant DDX3X allele (Raji, DG75, Daudi, GA10) (Supplementary Figure S1A). Most of these cell lines have upregulated DDX3Y possibly as a compensatory mechanism to counteract the loss of DDX3X function (Supplementary Figure S1B). Additionally, of the three female cell lines, one carried a wild-type DDX3X allele (Namalwa) and two carried a single mutant allele (EB1, ST486) (Supplementary Figure S1).

**FIGURE 1:**
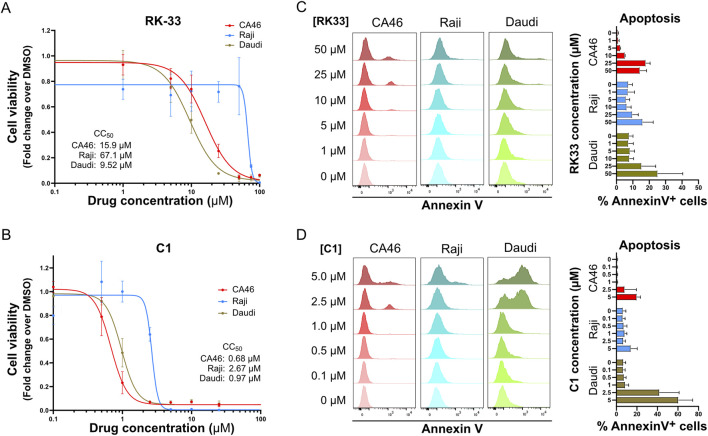
DDX3X inhibitors induce dose-dependent apoptosis in BL cell lines CA46, Raji and Daudi. **(A,B)** The dose-dependent effect of RK-33 **(A)** and C1 **(B)** on cell viability was assessed by XTT assay following 4 days of treatment with increasing concentrations of drugs. The calculated CC_50_ values for each cell line are indicated in the graphs. **(C,D)** Apoptosis was measured by flow cytometry of Annexin V staining and PI exclusion after 24 h of treatment with increasing concentrations of RK-33 **(C)** or C1 **(D)**. Results are expressed as the percentage of Annexin V^+^ cells. Bars represent mean ± SD from three independent experiments.

All BL cell lines exhibited varying degrees of sensitivity to DDX3X inhibition, including those that lack functional DDX3X and rely on DDX3Y for survival (Figures 1A,B; Supplementary Figure S2). Among them, Daudi, in which DDX3X was undetectable but DDX3Y was present at high levels (Supplementary Figure S1B), was highly sensitive to inhibition (Figure 1A). In contrast, Raji, which expresses a mutated DDX3X protein lacking helicase activity and relies on DDX3Y expression for survival (Wang et al., 2015) (Supplementary Figure S1B), was the most resistant (Supplementary Figure S1A). The CA46 cell line, which expresses wild-type DDX3X but no DDX3Y due to loss of the Y chromosome, displayed intermediate sensitivity to the inhibitors (Figure 1A; Supplementary Figure S1B).

Notably, the effects of DDX3X inhibition in Daudi, Raji, and CA46 BL cell lines were rapid, with detectable apoptosis occurring within 24 h at concentrations near the CC_50_ (Figures 1C,D). In these experiments, C1 demonstrated an order of magnitude higher cytotoxicity than RK-33, highlighting that more potent DDX3X inhibitors can effectively kill BL cell lines *in vitro*.

### RK-33 impedes progression of human Burkitt lymphoma in xenografts

**FIGURE 2:**
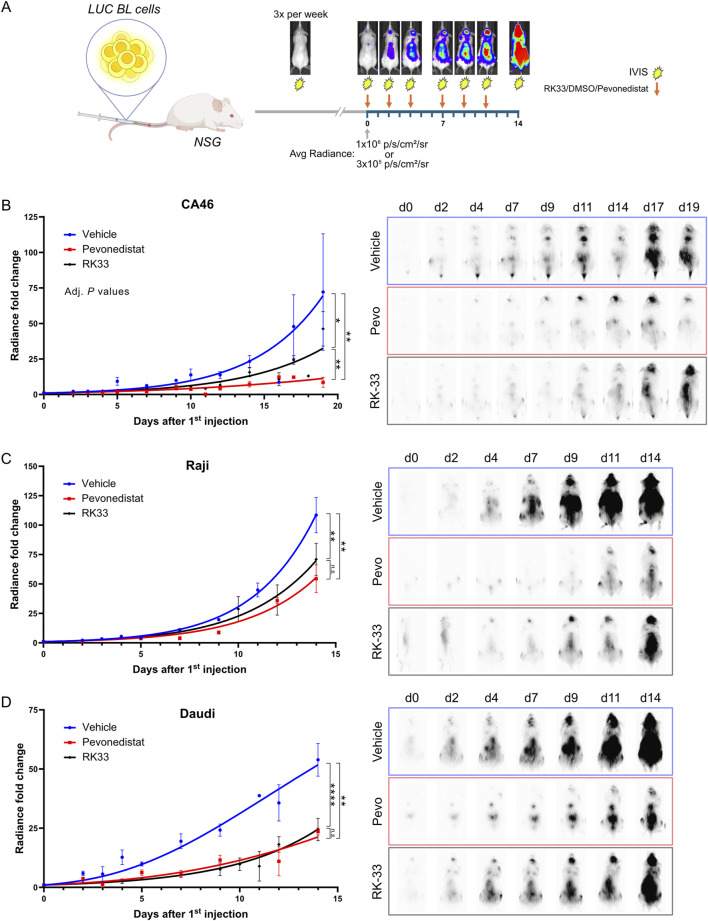
RK-33 decreases tumor growth rate in a disseminated Burkitt lymphoma xenograft model. **(A)** Dissemination model. To assess effect of DDX3X inhibition in a dissemination model that mimicks Burkitt lymphoma, NSG mice were *i.v.* injected with 5 × 10^5^ Fluc-CA46-Hygro, 2.5 × 10^5^ Fluc-Daudi-Puro or 1 × 10^5^ Fluc-Raji-Puro cells. Tumor progression was monitored every other day using an IVIS 200 Xenogen device to detect bioluminescence. Once the average radiance reached the indicated thresholds, mice received six doses of RK-33 over a 2-week period (arrows) while bioluminescent monitoring continued throughout. **(B–D)** Tumor dissemination progression over time following the initiation of the RK-33 treatment (Day 0 = first injection) in xenografts derived from CA46-Fluc-Hygro **(B)**, Raji-Fluc-Puro **(C)** and Daudi-Fluc-Puro **(D)** cells. Representative RAW bioluminescence images are shown with exposure times of 30 s for Fluc-CA46-Hygro and 1 s exposition for Raji-Fluc-Puro and Daudi-Fluc-Puro xenografts. are shown. *P < 0.05; **P < 0.01; ****P < 0.0001.

DDX3X inhibition effectiveness *in vivo* was evaluated through a dissemination model which emulates human Burkitt lymphoma (BL) (Figure 2A) (Matis et al., 2009). Immunodeficient NSG mice received intravenous injections with luciferase-expressing CA46 (Fluc-CA46-Hygro), Daudi (Fluc-Daudi-Puro), or Raji (Fluc-Raji-Puro) BL cells and were then monitored for tumor progression using an IVIS 200 Xenogen device to detect bioluminescence. Mice received RK-33 treatment after their tumor burden reached defined threshold values of 6 × 10^6^ photons/s/cm^2^/sr for Fluc-Raji-Puro and Fluc-Daudi-Puro cells and 3 × 10^5^ photons/s/cm^2^/sr for Fluc-CA46-Hygro cells before being monitored for 2 weeks (Figures 2A–D). We chose RK-33 instead of C1 because RK-33 had previously been used in cancer xenograft models (Wilky et al., 2016; Tantravedi et al., 2019) and C1 displayed poor bioavailability (data not shown). For comparison, an independent cohort of mice received Pevonedistat as a reference drug because it represents a potent new B-cell lymphoma treatment undergoing clinical trials that targets Neddylation, a protein modification similar to ubiquitination (Czuczman et al., 2016; Paiva et al., 2017; Torka et al., 2020) (Figures 2B–D).

Mice injected with the mildly aggressive wild-type DDX3X-expressing Fluc-CA46-Hygro cells demonstrated substantial responsiveness to Pevonedistat treatment and a moderate sensitivity to RK-33 compared to the vehicle-treated group (Figure 2B) even at near-maximal tolerated dose. Mice injected with the highly aggressive Fluc-Raji-Puro cells, which express mutated DDX3X and functional DDX3Y showed only a modest response to both Pevonedistat and RK-33 treatments. As seen with the CA46 model, Pevonedistat demonstrated superior effectiveness in impeding tumor progression than RK-33 (Figure 2C). However, the overall reduction in tumor burden for both treatments was marginal, resulting in only 2–3 days of extended survival time. This suggested that the mutant DDX3X in Raji cells may still contribute to tumorigenicity while remaining unaffected by RK-33 since its catalytic activity is already inactive, or that DDX3Y retains some residual activity in the presence of the inhibitor.

Interestingly, the RK-33 treatment of mice injected with Fluc-Daudi-Puro cells, which exhibit an intermediate level of aggressiveness compared to the CA46 and Raji cell lines, resulted in tumor suppression similar to Pevonedistat (Figure 2D). Since the survival of Daudi cells depends on DDX3Y overexpression because DDX3X expression is undetectable, these results suggest RK-33 is also effective in blocking DDX3Y to slow down tumor progression and that inhibition of both DDX3 paralogues can contribute to tumor suppression. The results also show that RK-33 slowed down tumor progression across all three BL models, but that Pevonedistat proved more effective which suggests the cell lines developed alternative survival mechanisms against DDX3X inhibition *in vivo*.

### RK-33 induces cellular stress responses with variable effects across cell lines

**FIGURE 3:**
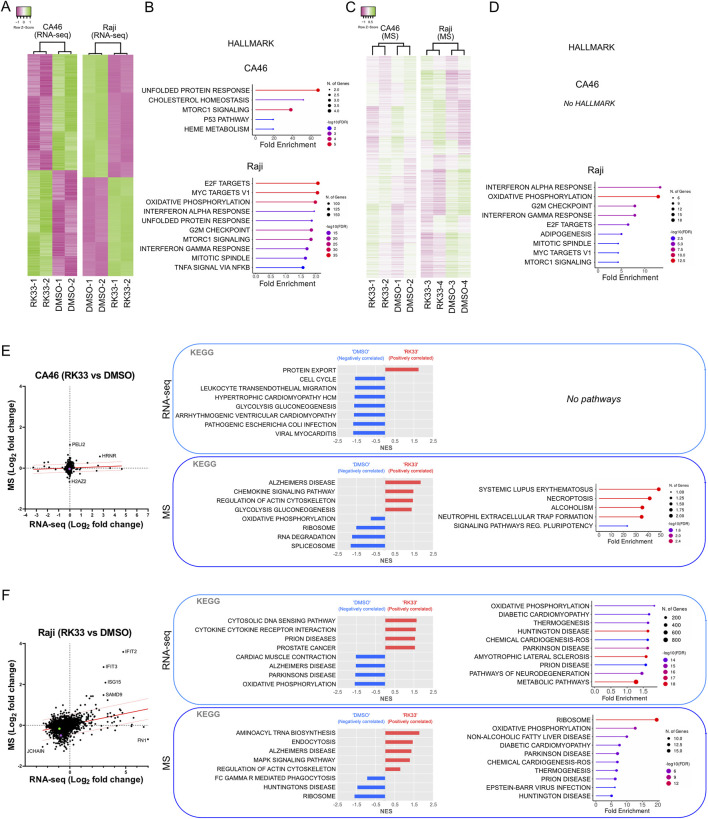
Effect of DDX3X inhibition by RK-33 on transcriptome and proteome of CA46 and Raji cell lines. **(A)** Hierarchical clustering heatmaps of RNA-seq data showing differential gene expression in CA46 (279 genes) and Raji (1000 top genes) cells treated with RK-33 and DMSO control (n = 2 per group). **(B)** Hallmark pathways identified through ShinyGO analysis (False discovery rate, FDR <0.05) on RNA-seq data: CA46 (17 genes) and Top 10 of 42 enriched pathways in Raji (13,958 genes). **(C)** Hierarchical clustering heatmaps of proteomic data that were normalized to account for the batch effect of the tandem mass tag (TMT) sets from CA46 (1137 peptides, average abundance >50) and Raji (1010 peptides, average abundance >50) following RK-33 or DMSO treatment. Samples were grouped into TMT sets as follows: CA46 is TMT-1 (DMSO-1 and RK33-1) and TMT-2 (DMSO-2 and RK33-2); Raji is TMT-3 (DMSO-3 and RK33-3) and TMT-4 (DMSO-4 and RK33-4)]. The clustering dendrograms of normalized data shows that samples cluster based on their treatment. **(D)** Hallmark pathways identified through ShinyGO analysis (FDR <0.05) based on peptides with fold change >1.4 in RK-33-treated cells relative to DMSO-treated controls: CA46 (8 peptides) and Raji (166 peptides). **(E–F)** Correlation plots of RNA expression and protein abundance in CA46 **(E)** and Raji **(F)** cells. KEGG gene set enrichment analysis (GSEA, left panel in each boxed section) and ShinyGO pathways (right panel) for RNA-seq (top box) and proteomic (MS) (botom box) datasets.

To identify changes in cellular pathways caused by DDX3X inhibition, we treated Raji cells and CA46 cells with the DDX3X inhibitor RK-33. The Raji cell line, which carries a catalytically inactive DDX3X and relies on wild-type DDX3Y was chosen because it represents a typical case of Burkitt lymphoma, in which a mutant DDX3X is present alongside DDX3Y, both potentially contributing to the malignancy. The CA46 cell line was selected because it expresses only wild-type DDX3X and lacks DDX3Y, therefore providing a simplified model and a control. These cell lines represent a range of situations found in human BL. Cells were treated for 24 h and then total RNA and protein were extracted for next-generation RNA sequencing (RNA-seq, Figures 3A,B) and Tandem Mass Tag mass spectrometry (TMT-MS, Figures 3C,D) respectively. Because the TMT-MS data produced a strong batch effect between the different TMT sets, as highlighted by the strong clustering between TMTs (Supplementary Figure S3A), data were normalized to account for this effect, allowing to assess the treatment-specific effects by minimizing the influence of TMT-specific variation (Figure 3C).

The results revealed cell line-specific responses to RK-33 treatment at both the transcriptional and proteomic levels. The CA46 cells showed a very limited number of differentially expressed transcripts after RK-33 treatment, indicating that the transcriptional changes were relatively small (Figure 3E). Similar to the transcriptomic data, proteomic profiling revealed very little disruption with only a small number of proteins having altered abundance (Figure 3E). In contrast, Raji cells, which were more resistant to RK-33 at the viability level, had a much broader response RK-33 treatment affected thousands of genes as shown by RNA-seq, and mass spectrometry also revealed widespread proteomic changes (Figure 3F).

To obtain further understanding of the biological pathways affected by RK-33, we used Gene Set Enrichment Analysis (GSEA) and ShinyGO for pathway enrichment analysis. These analyses identified different patterns of pathway regulation between the 2 cell lines. CA46 cells showed minimal pathway enrichment with only a few categories showing some marginal dysregulation (Figures 3B,D,E). On the other hand, Raji cells had robust enrichment in a wide range of biological pathways at both the mRNA and protein levels (Figures 3B,D,F).

Although there was little overlap of the differentially expressed genes or proteins between the 2 cell lines and between transcriptomic and proteomic datasets within the same cell line, several biological functions were repeatedly identified between the different conditions. Of those, protein metabolism (protein export, ribosome function, unfolded protein response), oxidative stress (oxidative phosphorylation, mTORC1 signaling, ROS related pathways), RNA processing and degradation, and cell cycle regulation (G2/M checkpoint, mitotic spindle) were some of the pathways commonly affected (Figures 3E,F). This indicated that RK-33-induced DDX3X inhibition disrupts general cellular homeostasis rather than disrupting specific molecular pathways.

These results confirmed that RK-33 likely acts, at least in part, through the induction of cellular stress responses that may eventually result in apoptosis. Furthermore, the data suggested that transcriptional dysregulation precedes proteomic alterations, suggesting that transcriptional reprogramming is among the earliest detectable cellular responses to DDX3X inhibition. This is consistent with the observation that despite the high transcriptional effects observed in Raji cells, the proteomic variability was important, even between biological replicates, which might suggest a stochastic and dynamic nature of the downstream responses to cellular stress (Figure 3C).

Taken together these results indicated that RK-33 has biological effects that are highly context dependent. The observed differential sensitivity and variability in pathway responses among BL cell lines suggested a complex, multifaceted role of DDX3X in maintaining cellular function and the importance of cellular context in determining therapeutic efficacy.

### siRNA knockdown of DDX3X/DDX3Y increases cellular stress, particularly through reactive oxygen species (ROS)

To determine whether any of the effects described above could be attributed to non-specific effects of RK-33, we conducted siRNA to knockdown DDX3X, DDX3Y, or both genes at the same time. This strategy not only allowed us to compare the results of specific gene silencing to those observed with pharmacological inhibition, but also to explore the contribution of wild-type DDX3X (in CA46) and the catalytically inactive DDX3X variant (in Raji). This was important because, some studies had shown that DDX3X may exhibit some cellular functions that do not depend on its RNA-helicase activity (Soulat et al., 2008; Geissler et al., 2012; He et al., 2024; Owens et al., 2024). Therefore, we conducted knockdown experiments to help distinguish between phenotypes that arise primarily from helicase inhibition and those caused by the loss of the protein.

**FIGURE 4:**
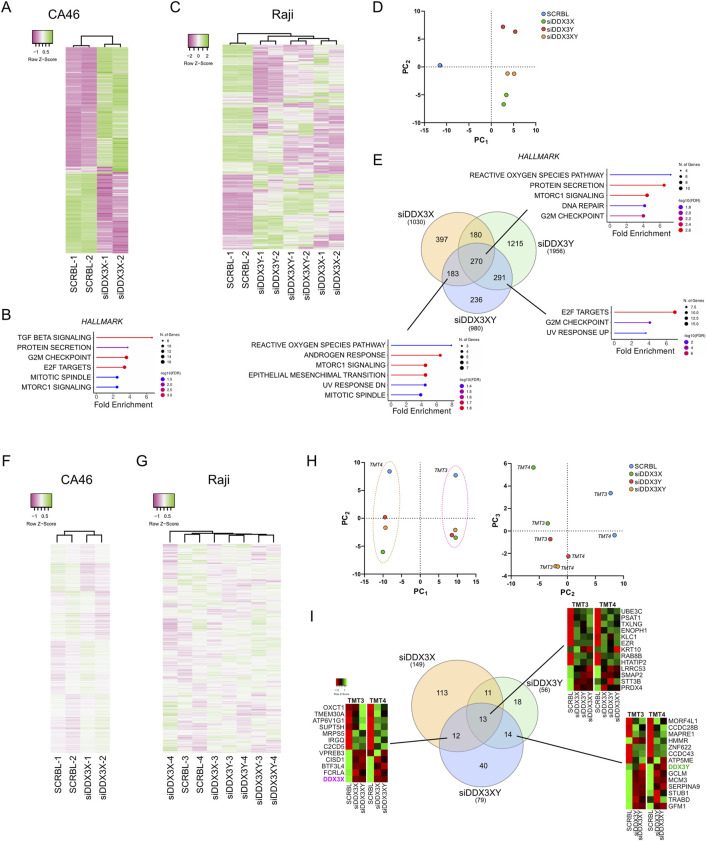
Effect of DDX3X and DDX3Y siRNA-mediated knockdown on transcriptome and proteome of CA46 and Raji cell lines. **(A)** Hierarchical clustering heatmap showing gene expression profiles (top 359 genes) from RNA-seq analysis of CA46 cells treated with siRNA targeting DDX3X (siDDX3X) or scrambled control (SCRBL) (n = 2 per group). **(B)** Hallmark pathways enriched in CA46 cells (524 differentially expressed genes, adjusted *P* < 0.05) following DDX3X knockdown, as identified by ShinyGO analysis (FDR <0.05). **(C)** Hierarchical clustering heatmap of RNA-seq data from Raji cells (top 782 genes) treated with siRNA targeting DDX3X (siDDX3X), DDX3Y (siDDX3Y), both (siDDX3XY), or scrambled control (SCRBL) (n = 2 per group). **(D)** Principal component analysis (PCA) of the full transcriptome in Raji cell treated with siRNAs as in **(C)**. PCA Component 1 (PC1) and 2 (PC2) show that all knockdown conditions cluster distinctly from scrambled controls along PC1, with siDDX3X clustering more closely with siDDX3XY than with siDDX3Y alone. **(E)** Hallmark pathways enriched in Raji cells following siRNA treatment, identified using ShinyGO (FDR <0.05). The number of differentially expressed genes per condition is indicated in the Venn diagram. **(F,G)** Hierarchical clustering heatmaps showing the distribution profiles of proteomic data that were normalized to account for the batch effect of the tandem mass tag (TMT) sets in CA46 **(F)** (961 peptides, average abundance >50) and Raji **(G)** (887 peptides, average abundance >50) cells treated with siRNAs as in **(A,C)**. TMT groups were assigned as follows: CA46 is TMT-1 (SCRBL-1 and siDDX3X-1) and TMT-2 (SCRBL-2 and siDDX3X-2); Raji is TMT-3 (SCRBL-3, siDDX3X-3, siDDX3Y-3 and siDDX3XY-3) and TMT-4 (SCRBL-4, siDDX3X-4, siDDX3Y-4 and siDDX3XY-4). The clustering dendrograms of normalized data shows that samples cluster based on their respective treatment. **(H)** PCA of the full proteome in Raji cell. PCA component 1 (PC1) and 2 (PC2) revealed a strict clustering effect along PC1 due to TMT grouping. Component 2 (PC2) and 3 (PC3) reveals limited clustering between knockdowns and between replicates. **(I)** Venn diagram showing the number of peptides changed in the different conditions. No hallmark pathways could be identified from the overlapping sets.

Since CA46 cells lack a Y chromosome, we only knocked down DDX3X in this line. However, Raji cells carry both a catalytically inactive DDX3X and the wild-type DDX3Y, thus enabling us to determine the functional significance of each variant by depleting them separately or in combination. The cells were transfected with siRNAs for 24 h before RNA-seq and TMT-based mass spectrometry analyses (Figure 4; Supplementary Figure S4). Importantly, both RNA-seq and mass spectrometry confirmed the successful knockdown of target genes on the RNA and protein levels but showed that siDDX3X was more efficient than siDDX3Y (Supplementary Figure S4). RNA-seq data showed that siRNA-mediated knockdown resulted in gene expression changes that were generally less pronounced than those seen with RK-33 treatment (Figures 4A,C). In both CA46 (Figure 4B) and Raji (Figure 4C) cells, only a small number of hallmark pathways were altered, and the changes in proteomics were even smaller (Figures 4F,G). Nonetheless, in Raji cells, PCA analysis of the transcription profiles showed that cells treated with siDDX3X alone or with both siDDX3X/DDX3Y were more similar to each other than to the scramble control or siDDX3Y alone (Figure 4D). Suggesting that the catalytically inactive DDX3X in Raji makes a substantial contribution to the transcriptome, pointing to a non-helicase-dependent functional role in these cells.

Although transcriptional changes were not massive, there was little overlap in differentially expressed genes between the different knockdowns in Raji cells (Figure 4E). However, using ShinyGO for gene set enrichment analysis, we observed a consistent enrichment in stress-related pathways, especially those related to the generation of reactive oxygen species and oxidative stress such as the MTORC1 Signaling hallmark (Figure 4E) which was also enriched in RK-33-treated cells along the oxydative phosphorylation hallmark. Moreover, ShinyGO analysis of the genes overlapping between the three knockdowns and RK-33-treated cells identified the same oxydative stress-related hallmarks with the reactive oxygen species pathway being the most enriched (Supplementary Figure S5). Broader pathway enrichment analyses across datasets also identified stress as a common theme, especially with respect to oxidative phosphorylation, protein metabolism, and cell cycle regulation (Supplementary Figure S4). These observations suggest that the knockdown of DDX3X or DDX3Y by siRNA causes a disruption of cellular homeostasis and activates a general stress response.

As observed in the proteomic data of RK-33-treated cells, the TMT-MS data produced a strong batch effect between the different TMT sets of siRNA-treated cells (Supplementary Figure S3B,C), and data were thus normalized to account for this effect (Figures 4F,G). At the proteomic level, siRNA knockdown had an even milder effect. Only a handful of proteins were differentially expressed across multiple knockdowns (Figures 4F–I). Importantly, PCA confirmed the heatmap clustering indicating that the main source of variance between proteomic datasets was not due to biological effects of knockdowns but rather due to technical aspects such as separating biological replicates into different TMT batches (Figure 4H). In both heatmap clustering of normalized data and PCA after removing the TMT batch effect by analyzing the components PC2 and PC3, the DDX3Y knockdown alone appeared to cluster closer to the dual knockdown than to the DDX3X knockdown alone, which shows a larger variation between two replicates than the other sets (Figures 4G,H). ShinyGO analysis of dysregulated peptides that are shared by different knockdowns did not identify any specific pathway enrichment, suggesting that the few observed alterations in protein levels may be random rather than specific biological responses (Figure 4I).

Despite proteomic changes that were very small and limited replicate clustering, the most marked effects observed when using broader gene set enrichment analyses were seen in Raji cells following the knockdown of the catalytically inactive DDX3X (Supplementary Figure S4B). This implies that despite the fact that the mutant DDX3X is inactive enzymatically, it still has a regulatory role in this particular case, possibly even more so than the wild-type DDX3Y. However, it is also possible that the greater impact of DDX3X knockdown may be due to the more efficient silencing of DDX3X than of DDX3Y.

In summary, depleting DDX3X and DDX3Y using siRNA recapitulated partially the transcriptional changes caused by inhibition of DDX3 by RK-33 with the induction of stress-related gene expression signatures. These data further support a model in which a catalytically inactive mutant forms of DDX3X may have non-canonical roles in Burkitt lymphoma.

### RK-33-mediated inhibition of DDX3X increases ROS and sensitizes cells to oxidative stress

**FIGURE 5:**
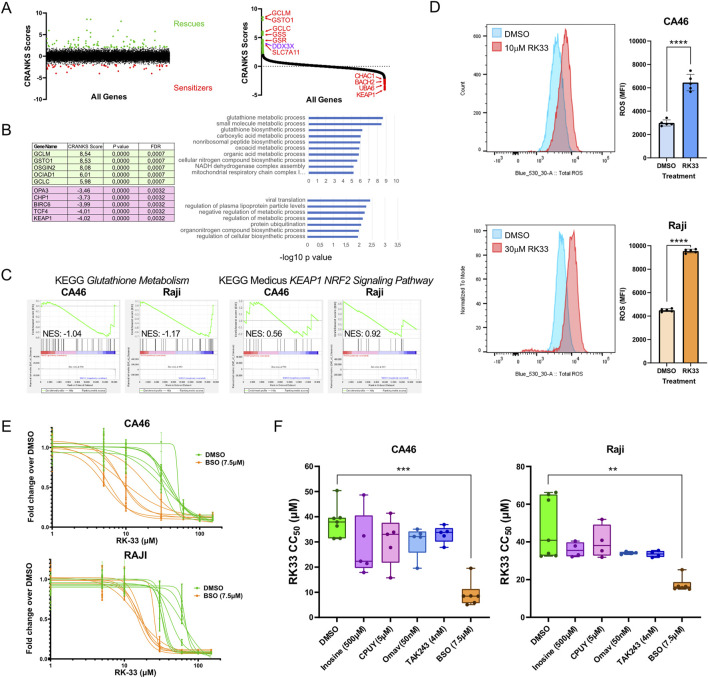
CRISPR chemogenomic screen identifies modulators of sensitivity and resistance to DDX3X inhibition by RK-33. **(A)** unranked CRANKS scores for all sgRNA (left) and ranked CRANKS score (right) from highest to lowest, showing sgRNA enrichment (rescues, green) and depleted (sensitizers, red) in RK-33-treated NALM-6 cells. sgRNAs targeting genes known to be involved in oxidative stress response are identified. **(B)** top 5 enriched (green) and top 5 depleted (pink) sgRNAs are shown, alongside Gene Ontology (GO) analysis for all significantly enriched (top chart) and depleted (bottom chart) sgRNAs. **(C)** Gene set enrichment analysis (GSEA) for the *KEGG Gluthatione Metabolism* (left) and *KEAP1-NRF2 signaling pathway* (right) using RNA-seq data from CA46 and Raji cells treated with RK-33 (as presented in Figure 3). **(D)** Total Reactive Oxygen Species (ROS) in CA46 and Raji cells after 24 h of treatment with DMSO or RK-33, measured by flow cytometry using the FITC channel and reported as mean fluorescence intensity (MFI). Statistical significance was calculated using unpaired *t*-tests. **(E,F)** Dose-dependent effects of combinatorial treatments using RK-33 and inhibitors of key hits from **(B)**, assessed by XTT viability assay after 4 days. CPUY192018 (CPUY) and omaveloxolone (Omav) are KEAP1 inhibitors; inosine and TAK243 are UBA6 inhibitors; and buthionine sulfoximine (BSO) is a GCL inhibitor. **(E)** Viability curves of cells treated with BSO in combination with RK-33. **(F)** Calculated CC_50_ values for each cell line and treatment combination, shown as box-and-whisker plots. *P* values were evaluated using a Kruskal–Wallis *H* test followed by Dunn’s multiple comparisons test comparing each combination treatment to vehicle + RK-33. In all graphs, **P < 0.01; ***P < 0.001; ****P < 0.0001.

To identify modulators of sensitivity or resistance to DDX3X inhibition by RK-33, a genome-wide CRISPR/Cas9 chemogenomic screen was conducted in NALM-6 cells, a human pre-B acute lymphoblastic leukemia cell line for which the screening protocol had been previously established (Bertomeu et al., 2018). This screen revealed a range of genes whose knockout either rescued cells from the effects of RK-33 or rendered them more sensitive to it (Figure 5A). Notably, DDX3X itself emerged as a “rescuer” hit, suggesting that complete loss of the gene can mitigate the cytotoxic impact of its pharmacological inhibition. This finding further supported our hypothesis that catalytically inactive DDX3X may retain abnormal functions within the cell, the loss of which reduces the detrimental effects induced by RK-33. Among the most significant rescue hits were several genes involved in the glutathione metabolic pathway, including *GCLM*, *GSTO1*, *GCLC*, *GSS*, and *GSR* (Figures 5A,B) and an ontology analysis of the top hits from the rescue group confirmed the enrichment of glutathione-related pathways (Figure 5B), which are central to the regulation of intracellular reactive oxygen species (ROS).

To further explore whether glutathione metabolism is altered upon RK-33 treatment, we re-examined RNA sequencing data from RK-33-treated Raji and CA46 BL cell lines. Gene set enrichment analysis using the KEGG glutathione metabolism pathway, revealed that this gene set was negatively correlated with the RK-33 treatment compared to DMSO controls (Figure 5C). This effect was particularly pronounced in Raji cells, which showed a greater sensitivity to RK-33 than the CA46 cells. These observations suggested that RK-33 may increase oxidative stress in BL cells through disruption of glutathione metabolism.

To investigate whether RK-33 directly induces ROS accumulation, we treated Raji and CA46 cells with RK-33 for 24 h at concentrations near their respective CC_50_ values and quantified intracellular ROS levels using flow cytometry. Both cell lines displayed a significant increase in total ROS following treatment, confirming that RK-33 induces oxidative stress in BL cells (Figure 5D). This increase in ROS is consistent with the involvement of redox imbalances in the cytotoxic mechanism of RK-33.

Given these findings, we next asked whether small molecule inhibitors targeting top screen hits could modulate the cellular response to RK-33. From the sensitizer group, we selected KEAP1 and UBA6, two genes for which pharmacological inhibitors are available. KEAP1, a central component of the Keap1-Nrf2-ARE pathway that regulates oxidative stress responses (Tu et al., 2019), was inhibited using CPUY192018 (5 µM)(Lu et al., 2019) and Omaveloxolone (50 nM)(Reisman et al., 2019). Despite its strong representation among synthetic lethal hits, co-treatment with either inhibitor and RK-33 did not result in a measurable synergy (Figure 5F; Supplementary Figure S6B,C). Similarly, inhibition of UBA6, an E1 ubiquitin-activating enzyme, using inosine (500 µM) (Zhang et al., 2022) and TAK-243 (4 nM) (Barghout et al., 2019), failed to produce any notable enhancement of RK-33 cytotoxicity in either cell line (Figure 5F; Supplementary Figure S6A,D).

We then focused on genes from the rescue group, particularly those involved in glutathione metabolism. Since several glutathione-related enzymes were identified as the most frequent rescuer hits in the screen, we tested whether inhibition of glutathione synthesis would affect RK-33 sensitivity. Cells were treated with Buthionine Sulfoximine (BSO, 7.5 µM), an inhibitor of glutamate-cysteine ligase (GCL), the first enzyme in glutathione biosynthesis (Griffith and Meister, 1979). Interestingly, whereas the chemogenomic screen in NALM-6 cells suggested that loss of glutathione pathway components would rescue RK-33 toxicity, treatment of Raji and CA46 cells with BSO resulted in a marked synthetic lethality when combined with RK-33 (Figures 5E,F). This discrepancy may be due to lineage-specific differences in redox buffering capacity and suggests that BL cells are dependent on the glutathione synthesis pathway to counterbalance RK-33-induced oxidative stress, whereas the NALM-6 cells might be relying more on the Keap1-Nrf2-ARE pathway to achieve the same results.

### RK-33 treatment induces cellular stress signatures in human primary B cells

**FIGURE 6:**
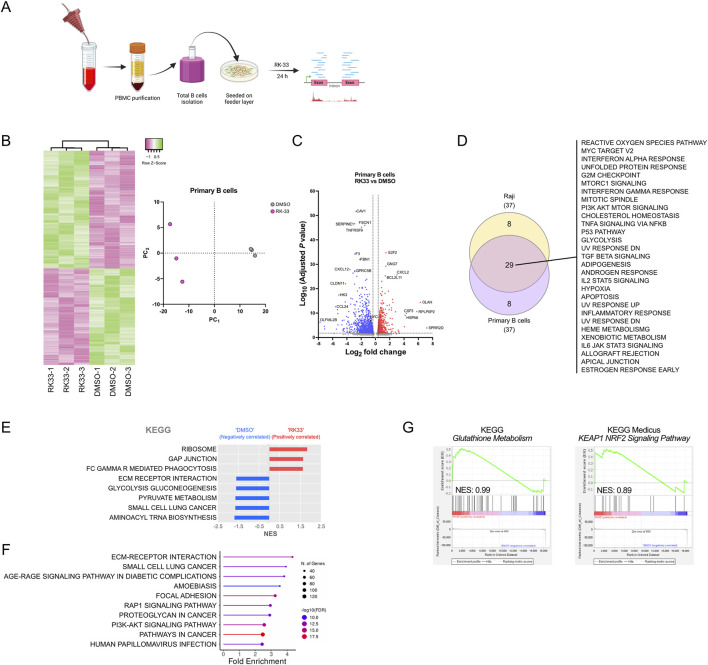
Effect of DDX3X inhibition by RK-33 on transcriptome of human primary B cells. **(A)** Scematic of primary B cell purification from periphera blood fom healty donors and subsequent treatment with RK-33 or DMSO control. **(B)** Hierarchical clustering heatmaps and principal component analysis (PCA) of the full transcriptome of RNA-seq data, showing differential gene expression in primary B cells (top 1000 most expressed genes) treated with RK-33 *versus* DMSO control (n = 3 per group). **(C)** Volcano plot of differentially expressed genes. With adjusted *P* < 0.05 and fold change >2 as thresholds, 522 genes were upregulated and 826 downregulated in RK-33-treated B cells compared to controls. **(D)** Venn diagram illustrating overlap of Hallmark pathways identified through ShinyGO analysis (FDR <0.05) of the 1,348 differentially expressed genes in RK-33-treated B cells and those identified in RK-33-treated Raji cells (Figure 3). **(E,F)** KEGG pathways enriched in RK-33-treated primary B cells relative to DMSO controls, as identified by GSEA **(E)** and a ShinyGO analysis **(F)**. **(G)** GSEA results for the KEGG *Gluthatione Metabolism* (left) and *KEAP1-NRF2 signaling pathway* (right) in primary B cells treated with RK-33.

To assess if DDX3X inhibition would affect normal B cells differently than BL cell lines, we used primary human B cells from peripheral blood of healthy donors who had undergone leukapheresis (Figure 6A). The RK-33 treatment produced widespread transcriptional alterations in primary B cells which showed similarities to Raji cell changes, but at reduced intensity (Figures 6B,C).

Hallmark analysis using ShinyGO showed that RK-33 treatment caused significant enrichment of stress-related hallmarks including oxidative stress responses together with cell cycle regulation and general protein metabolism, which showed substantial overlap with Raji cell results (Figure 6D). In addition, the KEGG pathways identified by GSEA and ShinyGO further supported an effect of general cellular stress programs (Figures 6E,F) with affected pathways appearing to be deregulated in a stochastic manner with no clear indication of selective targeting. This strongly suggested that the transcriptional changes may result from a general cellular destabilization rather than specific pathway inhibition.

Interestingly, although ROS-related pathways showed modest enrichment following RK-33 treatment, the glutathione metabolism pathway exhibited an opposite and much milder trend in primary B cells (Figure 6G) compared to BL cell lines. This suggested that the mechanisms leading to ROS accumulation in normal B cells upon RK-33 treatment might differ from those in malignant B cells despite oxidative stress being a common outcome of DDX3X inhibition in normal and malignant cells.

## Discussion

Although the current Burkitt lymphoma (BL) therapies can reach high remission rates, they are highly toxic and cause severe side effects including secondary cancers later in life, a significant burden for children (Sandlund and Martin, 2016; Egan et al., 2019; Abrahão et al., 2020). The findings of this study provide experimental evidence from mouse xenograft models that inhibitors of the RNA helicase DDX3X could represent promising avenues for the development of new treatments for BL. We have used both *in vitro* and *in vivo* models and have revealed some of the mechanistic underpinnings of DDX3X inhibition, which allow to suggest possible combinatorial approaches for treatment such as the combination of RK-33 with inhibitors of the glutathione synthesis pathway. Our study also provides insight into the cellular consequences of DDX3X inhibition by RK-33 in BL and normal B cells. Importantly, the differential responses observed between BL cells that carry DDX3X LOF alleles, express the WT form and that either express or lack the male paralogue DDX3Y underscore the complexity of the role of DDX3 helicases in cellular homeostasis and stress regulation in BL.

The evaluation of two DDX3 inhibitors (RK-33 and C1) showed their capacity to induce apoptosis in BL cell lines. Notably Daudi cells, with low DDX3X levels, were the most sensitive to inhibition, while Raji cells, which carry a mutated non-functional DDX3X, were more resistant, possible because they are relying on DDX3Y instead. This reinforces the importance of DDX3X in maintaining BL cell survival, thus making patients with functional DDX3X the most likely to benefit from targeted treatments. Additionally, the study confirms that C1 possesses higher cytotoxicity than RK-33 but is less promising because of a poorer bioavailability, indicating both the potential and importance of developing more potent DDX3X inhibitors that could be used at lower dosage in clinical applications.

RK-33 was less potent than Pevonedistat in CA46 and Raji cells, but had similar effects on Daudi cells, which underlined again the differential sensitivity of BL cells lines according to their mutational status of DDX3 alleles to pharmacological inhibition. Pevonedistat targets NEDD8 to disrupt protein homeostasis which blocks DNA repair pathways, thereby causing DNA strand break accumulation and cell death (Zhou et al., 2016; Torka et al., 2020). In contrast, RK-33 likely targets RNA-related functions of DDX3X to trigger G1 cell cycle arrest and apoptosis (Lai et al., 2010; Soto-Rifo and Ohlmann, 2013; Chen et al., 2015; Gong et al., 2021). Based on these distinct mechanisms, it was conceivable that combining RK-33 with Pevonedistat could have a synergistic effect against B-cell lymphoma. However, administration of both drugs, even at significantly reduced doses proved to be highly toxic, with all treated mice succumbing within 24 h. This unexpected lethality curtailed subsequent experimentation with drug combination.

The treatment of BL cells lines with RK-33 elicited distinct transcriptional and proteomic responses in CA46 and Raji cells. The transcriptional changes in Raji cells indicated that the presence of a catalytically inactive DDX3X mutant and the upregulation of DDX3Y results in heightened sensitivity to cellular stress signals. This would suggest that the male homologue DDX3Y, which is upregulated in Raji cells can only partially restore the function of DDX3X confirming previous findings (Lacroix et al., 2022a). The comparatively milder transcriptional response in CA46 cells, which expresses a functional DDX3X but lacks DDX3Y supports this view. It is also possible that RK-33-mediated inhibition of DDX3Y in the presence of the catalytically inactive form of DDX3X in Raji exacerbates the deleterious effect of the mutant protein. Indeed, inhibiting even a fraction of DDX3Y could lead to an accumulation of inactive DDX3 proteins (mutant DDX3X and inhibited DDX3Y), resulting in a more pronounced functional deficit than what is observed in CA46 cells, where only a fraction of functional DDX3X is inhibited. This idea that a non-functional DDX3X can have particularly severe consequences is further supported by the chemogenomic screening results that identified DDX3X loss as a genetic rescuer of RK-33-mediated inhibition. However, because Raji cells can tolerate higher doses of RK-33 than CA46 cells, and because cells were treated at doses below their respective CC_50_, it is also possible that the stronger effect observed in Raji could be due to the higher dose of inhibitor used. This possibility, indeed, raises the likelihood that at least some of the effects seen in Raji could be caused by some unspecific action of RK-33 that only come into effect at higher doses. Yet, the strong effects that RK-33 had in primary B cells, even at concentration comparable to that observed in CA46 cells, support the idea that most of the effect of RK-33 is due to specific inhibition of DDX3X rather than to potential unspecific targets.

Our proteomic analysis showed more modest changes than the transcriptomic analysis, suggesting that the impact of RK-33, at least during the first 24 h, is most effective at the level of mRNA transcription or stability, since both depend on DDX3. In addition, this difference aligns with previous reports indicating that DDX3 helicases influence both transcription and translation, potentially modulating stress responses via multiple pathways (Lee et al., 2008; Ko et al., 2014; Calviello et al., 2021; Rengarajan et al., 2025).

The siRNA knockdown experiments further highlighted the functional relevance of the catalytically inactive DDX3X mutant. In Raji cells, the knockdown of the DDX3X mutant alone produced stronger stress-related transcriptomic changes compared to DDX3Y knockdown. This supports other findings that even in its inactive form, DDX3X may either retain functions critical for stress response regulation or gain non-canonical functions (Valentin-Vega et al., 2016; Gong et al., 2021; Owens et al., 2024). These results also support the idea that the stronger response to RK-33 treatment in Raji cells than in CA46 cells is due to an accumulation of catalytically inactive DDX3. The inability of DDX3Y to fully compensate for DDX3X loss points to non-redundant roles of these helicases in maintaining cellular homeostasis, which is in accordance with other studies that have shown that the two paralogues exhibit differential activity through regulatory nanoscale RNA-protein clusters, and to distinctive liquid-liquid phase separation capacity (Shen et al., 2022; Yanas et al., 2024). Additionally, the observation from our chemogenomics screen that DDX3X loss can rescue RK-33-induced cell lethality supports the notion that the presence of a dysfunctional DDX3X mutant may in some cases exacerbate cellular stress through defects in protein synthesis (Brown et al., 2021; Mosti et al., 2025). This finding implies that therapeutic strategies targeting DDX3X should account for the specific mutational landscape of the tumor cells.

It has been shown before that inhibition of DDX3X can either lead to an increase or a decrease of oxidative stress such as ROS level depending on cellular or metabolic context (Heerma van Voss et al., 2018b; Liu et al., 2023; Luo et al., 2023). A significant outcome of the present study is the clear association between RK-33 treatment and oxidative stress in BL, as evidenced by increased ROS levels. The increased oxidative stress, particularly in Raji cells, suggested that DDX3 inhibition compromises the cellular antioxidant defense system through glutathione regulation, which is supported by other studies that have linked inhibition of DDX3X to ferroptosis, an iron-dependent cell death mechanism known to affect glutathione peroxidase and mitochondrial function (Mao et al., 2024; Dai et al., 2025). The observation that glutathione metabolism components modulate RK-33 sensitivity further emphasizes the role of ROS in the cytotoxic effects of DDX3X inhibition.

Intriguingly, the observation that closely related ROS-regulating pathways such as Keap1-Nrf2-ARE and glutathione metabolism were identified as both rescuer or sensitizer in the chemogenomic screen in the NALM-6 cells, and that inhibition of these modulators in BL output the opposite outcome in BL cell lines, seem to suggest that the cellular reaction to DDX3X-inhibition-induced ROS levels may be dependent on cellular context rather than a specific effect of DDX3X inhibition (Kannan et al., 2014). Importantly, the observation that inhibition of the glutathione synthesis pathway enhances RK-33-mediated cytotoxicity in BL cells, despite genes of this pathway being identified as rescuers in NALM-6 cells, may be explained by the particular sensitivity of BL cells to elevated ROS levels, a known characteristic of Burkitt lymphomas (Cerimele et al., 2005; Kawada et al., 2009). In addition, the Keap1-N rf2-ARE pathway is known to respond to ROS through regulation of the glutathione, but the inhibition of its main sensor Keap1 did not result in an increase of RK-33 potency. This was achieved only when glutathione synthesis was directly inhibited. This could suggest that either in BL glutathione is regulated by a pathway independent of Keap1-Nrf2-ARE such as the p38 MAPK pathway (Huseby et al., 2016), or that it is regulated through a Keap1-independent regulation of Nrf2 as it has been suggested before (Miao et al., 2005; Li et al., 2012). A possible explanation for this difference could lie in distinct regulatory mechanisms of the Warburg effect, where preferentially cells rely on aerobic glycolysis over oxidative phosphorylation, between BL cells and the pre-B ALL NALM-6 cell line (Mushtaq et al., 2015).

The observation that co-treatment with BSO, which depletes glutathione, significantly enhanced RK-33 cytotoxicity suggests a potential therapeutic strategy that exploits oxidative stress to maximize the impact of DDX3X inhibition in B-cell lymphoma. Importantly, BSO has already been shown to act synergistically with other drugs to enhance their efficacy in various cancers (Li et al., 2016; Zhao et al., 2019; Pereira et al., 2025). Altogether, our results indicate that oxidative stress is a key mediator of RK-33 cytotoxicity in BL cell lines and that this effect can be significantly amplified by targeting the glutathione antioxidant pathway. The differential outcomes observed across cell types also underscore the importance of cellular context when interpreting chemogenomic screening results (de Sa Junior et al., 2017).

The consistent induction of oxidative stress pathways in primary human B cells treated with RK-33 reinforces the universality of DDX3X’s role in the stress response regulation (Shih et al., 2012; Valentin-Vega et al., 2016; Gong et al., 2021; Luo et al., 2023). Because the extent of the response was lower in primary B cells compared to BL cells, our findings suggest that DDX3X inhibition could have broader applications in cancer therapy. Indeed, although potential cytotoxic effects on normal cells should be carefully considered, the higher oxidative stress response in BL could be exploited to target specifically cancer cells. Overall, our data provides a comprehensive understanding of the cellular consequences of DDX3X inhibition. They emphasize the therapeutic potential of targeting DDX3, also and perhaps particularly in tumors with DDX3X mutations and upregulation of DDX3Y and suggest that a combination of drugs targeting DDX3 with those that enhance oxidative stress may represent promising therapeutic avenues for human BL. However, further investigations into the downstream pathways regulated by DDX3X, will be necessary to refine such therapeutic strategies and minimize off-target effects. In summary, our findings indicate that DDX3X inhibition by RK-33 triggers a global stress response in both normal and malignant B cells, with oxidative stress emerging as a common consequence. However, the more pronounced transcriptional changes and involvement of antioxidant pathways in lymphoma cells suggest a differential sensitivity, which may underlie their increased vulnerability to DDX3X-targeted therapy.

## Data Availability

The datasets presented in this study can be found in online repositories. The names of the repository/repositories and accession number(s) can be found in the article/Supplementary Material.

## References

[B1] Abate F. Ambrosio M. R. Mundo L. Laginestra M. A. Fuligni F. Rossi M. (2015). Distinct viral and mutational spectrum of endemic burkitt lymphoma. PLoS Pathog. 11 (10), e1005158. 10.1371/journal.ppat.1005158 26468873 PMC4607508

[B2] Abrahão R. Ribeiro R. C. Brunson A. Keegan T. H. M. (2020). The burden of second primary cancers among childhood cancer survivors. Ann. Cancer Epidemiol. 4, 7. 10.21037/ace-2020-01

[B3] Asthana S. Martin H. Rupkey J. Patel S. Yoon J. Keegan A. (2022). The physiological roles of the exon junction complex in development and diseases. Cells 11 (7), 1192. 10.3390/cells11071192 35406756 PMC8997533

[B4] Babicki S. Arndt D. Marcu A. Liang Y. Grant J. R. Maciejewski A. (2016). Heatmapper: web-enabled heat mapping for all. Nucleic Acids Res. 44 (W1), W147–W153. 10.1093/nar/gkw419 27190236 PMC4987948

[B5] Barghout S. H. Patel P. S. Wang X. Xu G. W. Kavanagh S. Halgas O. (2019). Preclinical evaluation of the selective small-molecule UBA1 inhibitor, TAK-243, in acute myeloid leukemia. Leukemia 33 (1), 37–51. 10.1038/s41375-018-0167-0 29884901

[B6] Berletch J. B. Yang F. Xu J. Carrel L. Disteche C. M. (2011). Genes that escape from X inactivation. Hum. Genet. 130 (2), 237–245. 10.1007/s00439-011-1011-z 21614513 PMC3136209

[B7] Bertomeu T. Coulombe-Huntington J. Chatr-Aryamontri A. Bourdages K. G. Coyaud E. Raught B. (2018). A high-resolution genome-wide CRISPR/Cas9 viability screen reveals structural features and contextual diversity of the human cell-essential proteome. Mol. Cell. Biol. 38 (1), e00302-17. 10.1128/MCB.00302-17 PMC5730719 29038160

[B8] Bol G. M. Vesuna F. Xie M. Zeng J. Aziz K. Gandhi N. (2015a). Targeting DDX3 with a small molecule inhibitor for lung cancer therapy. EMBO Mol. Med. 7 (5), 648–669. 10.15252/emmm.201404368 25820276 PMC4492822

[B9] Bol G. M. Xie M. Raman V. (2015b). DDX3, a potential target for cancer treatment. Mol. Cancer 14, 188. 10.1186/s12943-015-0461-7 26541825 PMC4636063

[B10] Bouska A. Bi C. Lone W. Zhang W. Kedwaii A. Heavican T. (2017). Adult high-grade B-cell lymphoma with burkitt lymphoma signature: genomic features and potential therapeutic targets. Blood 130 (16), 1819–1831. 10.1182/blood-2017-02-767335 28801451 PMC5649549

[B11] Brown N. P. Vergara A. M. Whelan A. B. Guerra P. Bolger T. A. (2021). Medulloblastoma-associated mutations in the DEAD-Box RNA helicase DDX3X/DED1 cause specific defects in translation. J. Biol. Chem. 296, 100296. 10.1016/j.jbc.2021.100296 33460649 PMC7949108

[B12] Caeser R. Di Re M. Krupka J. A. Gao J. Lara-Chica M. Dias J. M. L. (2019). Genetic modification of primary human B cells to model high-grade lymphoma. Nat. Commun. 10 (1), 4543. 10.1038/s41467-019-12494-x 31586074 PMC6778131

[B13] Calviello L. Venkataramanan S. Rogowski K. J. Wyler E. Wilkins K. Tejura M. (2021). DDX3 depletion represses translation of mRNAs with complex 5' UTRs. Nucleic Acids Res. 49 (9), 5336–5350. 10.1093/nar/gkab287 33905506 PMC8136831

[B14] Cerimele F. Battle T. Lynch R. Frank D. A. Murad E. Cohen C. (2005). Reactive oxygen signaling and MAPK activation distinguish epstein-barr virus (EBV)-Positive *versus* EBV-Negative Burkitt's lymphoma. Proc. Natl. Acad. Sci. U. S. A. 102 (1), 175–179. 10.1073/pnas.0408381102 15611471 PMC544042

[B15] Chen H. H. Yu H. I. Chang J. J. Li C. W. Yang M. H. Hung M. C. (2024). DDX3 regulates cancer immune surveillance *via* 3' UTR-Mediated cell-surface expression of PD-L1. Cell. Rep. 43 (3), 113937. 10.1016/j.celrep.2024.113937 38489268

[B16] Chen H. H. Yu H. I. Cho W. C. Tarn W. Y. (2015). DDX3 modulates cell adhesion and motility and cancer cell metastasis *via* Rac1-mediated signaling pathway. Oncogene 34 (21), 2790–2800. 10.1038/onc.2014.190 25043297

[B17] Czuczman N. M. Barth M. J. Gu J. Neppalli V. Mavis C. Frys S. E. (2016). Pevonedistat, a NEDD8-activating enzyme inhibitor, is active in mantle cell lymphoma and enhances rituximab activity *in vivo* . Blood 127 (9), 1128–1137. 10.1182/blood-2015-04-640920 26675347 PMC4778163

[B18] Dai J. Z. Hsu W. J. Lin M. H. Shueng P. W. Lee C. C. Yang C. C. (2025). YAP-Mediated DDX3X confers resistance to ferroptosis in breast cancer cells by reducing lipid peroxidation. Free Radic. Biol. Med. 232, 330–339. 10.1016/j.freeradbiomed.2025.03.019 40089076

[B19] de Sa Junior P. L. Camara D. A. D. Porcacchia A. S. Fonseca P. M. M. Jorge S. D. Araldi R. P. (2017). The roles of ROS in cancer heterogeneity and therapy. Oxid. Med. Cell. Longev. 2017, 2467940. 10.1155/2017/2467940 29123614 PMC5662836

[B20] Ditton H. J. Zimmer J. Kamp C. Rajpert-De Meyts E. Vogt P. H. (2004). The AZFa gene DBY (DDX3Y) is widely transcribed but the protein is limited to the Male germ cells by translation control. Hum. Mol. Genet. 13 (19), 2333–2341. 10.1093/hmg/ddh240 15294876

[B21] Duan C. Lin X. Zou W. He Q. Wei F. Pan J. (2025). Targeting DDX3X eliminates leukemia stem cells in chronic myeloid leukemia by blocking NT5DC2 mRNA translation. Oncogene 44 (4), 241–254. 10.1038/s41388-024-03215-w 39516658

[B22] Egan G. Goldman S. Alexander S. (2019). Mature B-NHL in children, adolescents and young adults: current therapeutic approach and emerging treatment strategies. Br. J. Haematol. 185 (6), 1071–1085. 10.1111/bjh.15734 30613948

[B23] Elso C. M. Roberts L. J. Smyth G. K. Thomson R. J. Baldwin T. M. Foote S. J. (2004). Leishmaniasis host response loci (lmr1-3) modify disease severity through a Th1/Th2-independent pathway. Genes. Immun. 5 (2), 93–100. 10.1038/sj.gene.6364042 14668789

[B24] Ge S. X. Jung D. Yao R. (2020). ShinyGO: a graphical gene-set enrichment tool for animals and plants. Bioinformatics 36 (8), 2628–2629. 10.1093/bioinformatics/btz931 31882993 PMC7178415

[B25] Geissler R. Golbik R. P. Behrens S. E. (2012). The DEAD-Box helicase DDX3 supports the assembly of functional 80S ribosomes. Nucleic Acids Res. 40 (11), 4998–5011. 10.1093/nar/gks070 22323517 PMC3367175

[B26] Gong C. Krupka J. A. Gao J. Grigoropoulos N. F. Giotopoulos G. Asby R. (2021). Sequential inverse dysregulation of the RNA helicases DDX3X and DDX3Y facilitates MYC-Driven lymphomagenesis. Mol. Cell. 81 (19), 4059–4075.e11. 10.1016/j.molcel.2021.07.041 34437837

[B27] Gostissa M. Yan C. T. Bianco J. M. Cogne M. Pinaud E. Alt F. W. (2009). Long-range oncogenic activation of Igh-c-myc translocations by the igh 3' regulatory region. Nature 462 (7274), 803–807. 10.1038/nature08633 20010689 PMC2802177

[B28] Grande B. M. Gerhard D. S. Jiang A. Griner N. B. Abramson J. S. Alexander T. B. (2019). Genome-wide discovery of somatic coding and noncoding mutations in pediatric endemic and sporadic burkitt lymphoma. Blood 133 (12), 1313–1324. 10.1182/blood-2018-09-871418 30617194 PMC6428665

[B29] Griffith O. W. Meister A. (1979). Potent and specific inhibition of glutathione synthesis by buthionine sulfoximine (S-n-butyl homocysteine sulfoximine). J. Biol. Chem. 254 (16), 7558–7560. 10.1016/s0021-9258(18)35980-5 38242

[B30] He Y. N. Han X. R. Wang D. Hou J. L. Hou X. M. (2024). Dual mode of DDX3X as an ATP-Dependent RNA helicase and ATP-Independent nucleic acid chaperone. Biochem. Biophys. Res. Commun. 714, 149964. 10.1016/j.bbrc.2024.149964 38669753

[B31] Heerma van Voss M. R. Kammers K. Vesuna F. Brilliant J. Bergman Y. Tantravedi S. (2018a). Global effects of DDX3 inhibition on cell cycle regulation identified by a combined phosphoproteomics and single cell tracking approach. Transl. Oncol. 11 (3), 755–763. 10.1016/j.tranon.2018.04.001 29684792 PMC6050443

[B32] Heerma van Voss M. R. Vesuna F. Bol G. M. Afzal J. Tantravedi S. Bergman Y. (2018b). Targeting mitochondrial translation by inhibiting DDX3: a novel radiosensitization strategy for cancer treatment. Oncogene 37 (1), 63–74. 10.1038/onc.2017.308 28869602 PMC5756132

[B33] Huseby N. E. Ravuri C. Moens U. (2016). The proteasome inhibitor lactacystin enhances GSH synthesis capacity by increased expression of antioxidant components in an Nrf2-independent, but p38 MAPK-Dependent manner in rat colorectal carcinoma cells. Free Radic. Res. 50 (1), 1–13. 10.3109/10715762.2015.1100730 26530909

[B34] Joshi A. Hannah R. Diamanti E. Gottgens B. (2013). Gene set control analysis predicts hematopoietic control mechanisms from genome-wide transcription factor binding data. Exp. Hematol. 41 (4), 354–366. 10.1016/j.exphem.2012.11.008 23220237 PMC3630327

[B35] Kannan N. Nguyen L. V. Makarem M. Dong Y. Shih K. Eirew P. (2014). Glutathione-dependent and -independent oxidative stress-control mechanisms distinguish normal human mammary epithelial cell subsets. Proc. Natl. Acad. Sci. U. S. A. 111 (21), 7789–7794. 10.1073/pnas.1403813111 24821780 PMC4040592

[B36] Kawada J. Zou P. Mazitschek R. Bradner J. E. Cohen J. I. (2009). Tubacin kills epstein-barr virus (EBV)-burkitt lymphoma cells by inducing reactive oxygen species and EBV lymphoblastoid cells by inducing apoptosis. J. Biol. Chem. 284 (25), 17102–17109. 10.1074/jbc.M809090200 19386607 PMC2719348

[B37] Kaymaz Y. Oduor C. I. Yu H. Otieno J. A. Ong'echa J. M. Moormann A. M. (2017). Comprehensive transcriptome and mutational profiling of endemic burkitt lymphoma reveals EBV type-specific differences. Mol. Cancer Res. 15 (5), 563–576. 10.1158/1541-7786.MCR-16-0305 28465297 PMC5471630

[B38] Ko C. Lee S. Windisch M. P. Ryu W. S. (2014). DDX3 DEAD-Box RNA helicase is a host factor that restricts hepatitis B virus replication at the transcriptional level. J. Virol. 88 (23), 13689–13698. 10.1128/JVI.02035-14 25231298 PMC4248967

[B39] Lacroix M. Beauchemin H. Fraszczak J. Ross J. Shooshtarizadeh P. Chen R. (2022a). The X-Linked helicase DDX3X is required for lymphoid differentiation and MYC-driven lymphomagenesis. Cancer Res. 82 (17), 3172–3186. 10.1158/0008-5472.CAN-21-2454 35815807

[B40] Lacroix M. Beauchemin H. Khandanpour C. Moroy T. (2023). The RNA helicase DDX3 and its role in c-MYC driven germinal center-derived B-cell lymphoma. Front. Oncol. 13, 1148936. 10.3389/fonc.2023.1148936 37035206 PMC10081492

[B41] Lacroix M. Beauchemin H. Moroy T. (2022b). DDX3: a relevant therapeutic target for lymphoma? Expert Opin. Ther. Targets 26 (12), 1037–1040. 10.1080/14728222.2022.2166830 36620925

[B42] Lai M. C. Chang W. C. Shieh S. Y. Tarn W. Y. (2010). DDX3 regulates cell growth through translational control of cyclin E1. Mol. Cell. Biol. 30 (22), 5444–5453. 10.1128/MCB.00560-10 20837705 PMC2976371

[B43] Lee C. S. Dias A. P. Jedrychowski M. Patel A. H. Hsu J. L. Reed R. (2008). Human DDX3 functions in translation and interacts with the translation initiation factor eIF3. Nucleic Acids Res. 36 (14), 4708–4718. 10.1093/nar/gkn454 18628297 PMC2504307

[B44] Li Q. Yin X. Wang W. Zhan M. Zhao B. Hou Z. (2016). The effects of buthionine sulfoximine on the proliferation and apoptosis of biliary tract cancer cells induced by cisplatin and gemcitabine. Oncol. Lett. 11 (1), 474–480. 10.3892/ol.2015.3879 26870236 PMC4727028

[B45] Li Y. Paonessa J. D. Zhang Y. (2012). Mechanism of chemical activation of Nrf2. PLoS One 7 (4), e35122. 10.1371/journal.pone.0035122 22558124 PMC3338841

[B46] Liu Y. Zhang Y. Wang C. Liu Q. Li T. Wang W. (2023). Inhibition of DDX3X alleviates persistent inflammation, immune suppression and catabolism syndrome in a septic mice model. Int. Immunopharmacol. 117, 109779. 10.1016/j.intimp.2023.109779 36806038

[B47] Lopez C. Kleinheinz K. Aukema S. M. Rohde M. Bernhart S. H. Hubschmann D. (2019). Genomic and transcriptomic changes complement each other in the pathogenesis of sporadic burkitt lymphoma. Nat. Commun. 10 (1), 1459. 10.1038/s41467-019-08578-3 30926794 PMC6440956

[B48] Lu M. C. Zhao J. Liu Y. T. Liu T. Tao M. M. You Q. D. (2019). CPUY192018, a potent inhibitor of the Keap1-Nrf2 protein-protein interaction, alleviates renal inflammation in mice by restricting oxidative stress and NF-κB activation. Redox Biol. 26, 101266. 10.1016/j.redox.2019.101266 31279986 PMC6614503

[B49] Luo T. Yang S. Zhao T. Zhu H. Chen C. Shi X. (2023). Hepatocyte DDX3X protects against drug-induced acute liver injury *via* controlling stress granule formation and oxidative stress. Cell. Death Dis. 14 (7), 400. 10.1038/s41419-023-05913-x 37407573 PMC10322869

[B50] Mao Q. Ma S. Li S. Zhang Y. Li S. Wang W. (2024). PRRSV hijacks DDX3X protein and induces ferroptosis to facilitate viral replication. Vet. Res. 55 (1), 103. 10.1186/s13567-024-01358-y 39155369 PMC11331664

[B51] Matis S. Mariani M. R. Cutrona G. Cilli M. Piccardi F. Daga A. (2009). PNAEmu can significantly reduce Burkitt's lymphoma tumor burden in a SCID mice model: cells dissemination similar to the human disease. Cancer Gene Ther. 16 (10), 786–793. 10.1038/cgt.2009.26 19363465

[B52] Miao W. Hu L. Scrivens P. J. Batist G. (2005). Transcriptional regulation of NF-E2 p45-related factor (NRF2) expression by the aryl hydrocarbon receptor-xenobiotic response element signaling pathway: direct cross-talk between phase I and II drug-metabolizing enzymes. J. Biol. Chem. 280 (21), 20340–20348. 10.1074/jbc.M412081200 15790560

[B53] Miard S. Girard M. J. Joubert P. Carter S. Gonzales A. Guo H. (2017). Absence of Malat1 does not prevent DEN-Induced hepatocarcinoma in mice. Oncol. Rep. 37 (4), 2153–2160. 10.3892/or.2017.5468 28260109

[B54] Mo J. Liang H. Su C. Li P. Chen J. Zhang B. (2021). DDX3X: structure, physiologic functions and cancer. Mol. Cancer 20 (1), 38. 10.1186/s12943-021-01325-7 33627125 PMC7903766

[B55] Moffitt A. B. Dave S. S. (2017). Clinical applications of the genomic landscape of aggressive non-hodgkin lymphoma. J. Clin. Oncol. 35 (9), 955–962. 10.1200/JCO.2016.71.7603 28297626

[B56] Molyneux E. M. Rochford R. Griffin B. Newton R. Jackson G. Menon G. (2012). Burkitt's lymphoma. Lancet 379 (9822), 1234–1244. 10.1016/S0140-6736(11)61177-X 22333947

[B57] Mosti F. Hoye M. L. Escobar-Tomlienovich C. F. Silver D. L. (2025). Multi-modal investigation reveals pathogenic features of diverse DDX3X missense mutations. PLoS Genet. 21 (1), e1011555. 10.1371/journal.pgen.1011555 39836689 PMC11771946

[B58] Mushtaq M. Darekar S. Klein G. Kashuba E. (2015). Different mechanisms of regulation of the warburg effect in lymphoblastoid and burkitt lymphoma cells. PLoS One 10 (8), e0136142. 10.1371/journal.pone.0136142 26312753 PMC4551852

[B59] Nakao S. Nogami M. Iwatani M. Imaeda T. Ito M. Tanaka T. (2020). Identification of a selective DDX3X inhibitor with newly developed quantitative high-throughput RNA helicase assays. Biochem. Biophys. Res. Commun. 523 (3), 795–801. 10.1016/j.bbrc.2019.12.094 31954521

[B60] Owens M. C. Shen H. Yanas A. Mendoza-Figueroa M. S. Lavorando E. Wei X. (2024). Specific catalytically impaired DDX3X mutants form sexually dimorphic hollow condensates. Nat. Commun. 15 (1), 9553. 10.1038/s41467-024-53636-0 39500865 PMC11538506

[B61] Paiva C. Godbersen J. C. Rowland T. Danilova O. V. Danes C. Berger A. (2017). Pevonedistat, a Nedd8-activating enzyme inhibitor, sensitizes neoplastic B-cells to death receptor-mediated apoptosis. Oncotarget 8 (13), 21128–21139. 10.18632/oncotarget.15050 28177892 PMC5400571

[B62] Pereira J. C. de Sousa R. W. R. Conceicao M. L. P. do Nascimento M. de Almeida A. T. A. Dos Reis A. C. (2025). Buthionine sulfoximine acts synergistically with doxorubicin as a sensitizer molecule on different tumor cell lines. J. Toxicol. Environ. Health A 88 (10), 409–431. 10.1080/15287394.2024.2448663 39815616

[B63] Ramathal C. Angulo B. Sukhwani M. Cui J. Durruthy-Durruthy J. Fang F. (2015). DDX3Y gene rescue of a Y chromosome AZFa deletion restores germ cell formation and transcriptional programs. Sci. Rep. 5, 15041. 10.1038/srep15041 26456624 PMC4601010

[B64] Reisman S. A. Gahir S. S. Lee C. I. Proksch J. W. Sakamoto M. Ward K. W. (2019). Pharmacokinetics and pharmacodynamics of the novel Nrf2 activator omaveloxolone in Primates. Drug Des. Devel Ther. 13, 1259–1270. 10.2147/DDDT.S193889 PMC6475100 31118567

[B65] Rengarajan S. Derks J. Bellott D. W. Slavov N. Page D. C. (2025). Post-transcriptional cross- and auto-regulation buffer expression of the human RNA helicases DDX3X and DDX3Y. Genome Res. 35 (1), 20–30. 10.1101/gr.279707.124 39794123 PMC11789639

[B66] Richter J. Schlesner M. Hoffmann S. Kreuz M. Leich E. Burkhardt B. (2012). Recurrent mutation of the ID3 gene in burkitt lymphoma identified by integrated genome, exome and transcriptome sequencing. Nat. Genet. 44 (12), 1316–1320. 10.1038/ng.2469 23143595

[B67] Sandlund J. T. Martin M. G. (2016). Non-hodgkin lymphoma across the pediatric and adolescent and young adult age spectrum. Hematol. Am. Soc. Hematol. Educ. Program 2016 (1), 589–597. 10.1182/asheducation-2016.1.589 PMC6142492 27913533

[B68] Schmitz R. Young R. M. Ceribelli M. Jhavar S. Xiao W. Zhang M. (2012). Burkitt lymphoma pathogenesis and therapeutic targets from structural and functional genomics. Nature 490 (7418), 116–120. 10.1038/nature11378 22885699 PMC3609867

[B69] Shen H. Yanas A. Owens M. C. Zhang C. Fritsch C. Fare C. M. (2022). Sexually dimorphic RNA helicases DDX3X and DDX3Y differentially regulate RNA metabolism through phase separation. Mol. Cell. 82 (14), 2588–2603.e9. 10.1016/j.molcel.2022.04.022 35588748 PMC9308757

[B70] Shih J. W. Wang W. T. Tsai T. Y. Kuo C. Y. Li H. K. Wu Lee Y. H. (2012). Critical roles of RNA helicase DDX3 and its interactions with eIF4E/PABP1 in stress granule assembly and stress response. Biochem. J. 441 (1), 119–129. 10.1042/BJ20110739 21883093

[B71] Short N. J. Kantarjian H. M. Ko H. Khoury J. D. Ravandi F. Thomas D. A. (2017). Outcomes of adults with relapsed or refractory burkitt and high-grade B-cell leukemia/lymphoma. Am. J. Hematol. 92 (6), E114-E117–E117. 10.1002/ajh.24720 28295472 PMC5828013

[B72] Song H. Ji X. (2019). The mechanism of RNA duplex recognition and unwinding by DEAD-Box helicase DDX3X. Nat. Commun. 10 (1), 3085. 10.1038/s41467-019-11083-2 31300642 PMC6626043

[B73] Soto-Rifo R. Ohlmann T. (2013). The role of the DEAD-Box RNA helicase DDX3 in mRNA metabolism. Wiley Interdiscip. Rev. RNA 4 (4), 369–385. 10.1002/wrna.1165 23606618

[B74] Soulat D. Burckstummer T. Westermayer S. Goncalves A. Bauch A. Stefanovic A. (2008). The DEAD-Box helicase DDX3X is a critical component of the TANK-Binding kinase 1-dependent innate immune response. EMBO J. 27 (15), 2135–2146. 10.1038/emboj.2008.126 18583960 PMC2453059

[B75] Stringer B. W. Day B. W. D'Souza R. C. J. Jamieson P. R. Ensbey K. S. Bruce Z. C. (2019). A reference collection of patient-derived cell line and xenograft models of proneural, classical and mesenchymal glioblastoma. Sci. Rep. 9 (1), 4902. 10.1038/s41598-019-41277-z 30894629 PMC6427001

[B76] Tantravedi S. Vesuna F. Winnard P. T. Jr. Martin A. Lim M. Eberhart C. G. (2019). Targeting DDX3 in medulloblastoma using the small molecule inhibitor RK-33. Transl. Oncol. 12 (1), 96–105. 10.1016/j.tranon.2018.09.002 30292066 PMC6171097

[B77] Torka P. Mavis C. Kothari S. Belliotti S. Gu J. Sundaram S. (2020). Pevonedistat, a NEDD8-Activating enzyme inhibitor, induces apoptosis and augments efficacy of chemotherapy and small molecule inhibitors in pre-clinical models of diffuse large B-cell lymphoma. EJHaem 1 (1), 122–132. 10.1002/jha2.2 33073261 PMC7566777

[B78] Tu W. Wang H. Li S. Liu Q. Sha H. (2019). The anti-inflammatory and anti-oxidant mechanisms of the Keap1/Nrf2/ARE signaling pathway in chronic diseases. Aging Dis. 10 (3), 637–651. 10.14336/AD.2018.0513 31165007 PMC6538222

[B79] Valentin-Vega Y. A. Wang Y. D. Parker M. Patmore D. M. Kanagaraj A. Moore J. (2016). Cancer-associated DDX3X mutations drive stress granule assembly and impair global translation. Sci. Rep. 6, 25996. 10.1038/srep25996 27180681 PMC4867597

[B80] Wang T. Birsoy K. Hughes N. W. Krupczak K. M. Post Y. Wei J. J. (2015). Identification and characterization of essential genes in the human genome. Science 350 (6264), 1096–1101. 10.1126/science.aac7041 26472758 PMC4662922

[B81] Wilky B. A. Kim C. McCarty G. Montgomery E. A. Kammers K. DeVine L. R. (2016). RNA helicase DDX3: a novel therapeutic target in ewing sarcoma. Oncogene 35 (20), 2574–2583. 10.1038/onc.2015.336 26364611

[B82] Winnard P. T. Jr. Vesuna F. Bol G. M. Gabrielson K. L. Chenevix-Trench G. Ter Hoeve N. D. (2024). Targeting RNA helicase DDX3X with a small molecule inhibitor for breast cancer bone metastasis treatment. Cancer Lett. 604, 217260. 10.1016/j.canlet.2024.217260 39306228

[B83] Xie M. Vesuna F. Tantravedi S. Bol G. M. Heerma van Voss M. R. Nugent K. (2016). RK-33 radiosensitizes prostate cancer cells by blocking the RNA helicase DDX3. Cancer Res. 76 (21), 6340–6350. 10.1158/0008-5472.CAN-16-0440 27634756 PMC5576499

[B84] Yanas A. Shweta H. Owens M. C. Liu K. F. Goldman Y. E. (2024). RNA helicases DDX3X and DDX3Y form nanometer-scale RNA-Protein clusters that support catalytic activity. Curr. Biol. 34 (24), 5714–5727.e6. 10.1016/j.cub.2024.10.055 39591970 PMC11978499

[B85] Yang S. N. Y. Atkinson S. C. Audsley M. D. Heaton S. M. Jans D. A. Borg N. A. (2020). RK-33 is a broad-spectrum antiviral agent that targets DEAD-box RNA helicase DDX3X. Cells 9 (1), 170. 10.3390/cells9010170 31936642 PMC7016805

[B86] Zahnreich S. Schmidberger H. (2021). Childhood cancer: occurrence, treatment and risk of second primary malignancies. Cancers (Basel) 13 (11), 2607. 10.3390/cancers13112607 34073340 PMC8198981

[B87] Zhang L. Jiang L. Yu L. Li Q. Tian X. He J. (2022). Inhibition of UBA6 by inosine augments tumour immunogenicity and responses. Nat. Commun. 13 (1), 5413. 10.1038/s41467-022-33116-z 36109526 PMC9478149

[B88] Zhao Y. Tanaka S. Yuan B. Sugiyama K. Onda K. Kiyomi A. (2019). Arsenic disulfide combined with L-Buthionine-(S, R)-Sulfoximine induces synergistic antitumor effects in two-dimensional and three-dimensional models of MCF-7 breast carcinoma cells. Am. J. Chin. Med. 47 (5), 1149–1170. 10.1142/S0192415X19500599 31311297

[B89] Zhou L. Chen S. Zhang Y. Kmieciak M. Leng Y. Li L. (2016). The NAE inhibitor pevonedistat interacts with the HDAC inhibitor belinostat to target AML cells by disrupting the DDR. Blood 127 (18), 2219–2230. 10.1182/blood-2015-06-653717 26851293 PMC4859196

