# Supplementary material for: Inhibiting the RNA helicase DDX3X in Burkitt lymphoma induces oxydative stress and impedes tumor progression in xenografts
Source: Front Cell Dev Biol. 2025 Jul 23;13:1642006. doi: 10.3389/fcell.2025.1642006 (PMC12325266; doi:10.3389/fcell.2025.1642006)
Supplement: Supplementary file 3 [file DataSheet1.pdf]

Figure S1

A

Cell line	sex	DDX3X Mutations
CA46	♂	
Raji	♂	Splice Variant, in frame deletion (d320-342)
Daudi	♂	Missense mutation (N653S), Low expression
BL-41	♂	
Namalwa	♀	
GA-10	♂	In Frame Deletion (E566del)
DG-75	♂	Frame Shift Deletion (S492AfsTer4)
EB1	♀	Missense mutation (G302S)
ST486	♀	Frame Shift Deletion (M379DfsTer3)

B

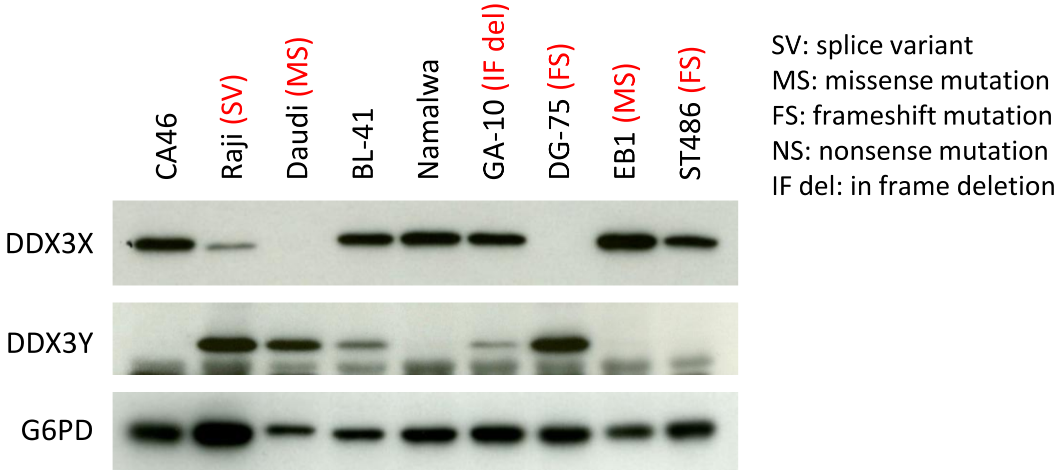

Supplementary Figure S1. Characterization of DDX3X and DDX3Y in several Burkitt lymphoma cell lines **(A)** Mutations in the gene of DDX3X in the BL cell lines obtained from the Broad depmap portal (https://depmap.org/portal) The 3 cell lines in blue are used in this study. **(B)** Western blot analysis of DDX3X and DDX3Y in all cell lines shown in **(A)**.

Figure S2

A

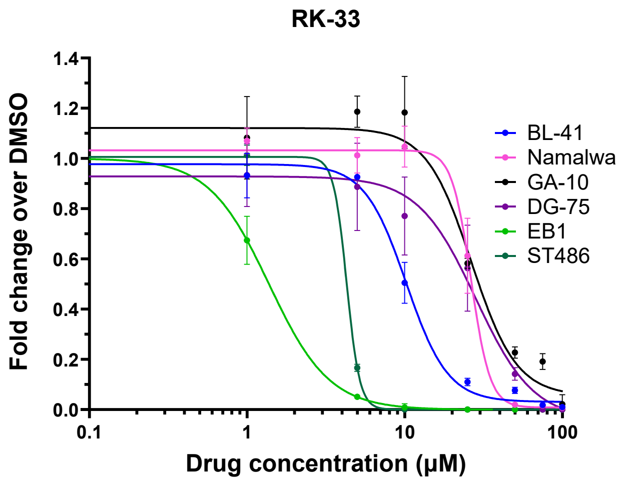

Cell line	CC_{50}
BL-41	10.23 µM
Namalwa	26.30 µM
GA-10	26.47 µM
DG-75	28.19 µM
EB1	1.38 µM
ST486	4.29 µM

B

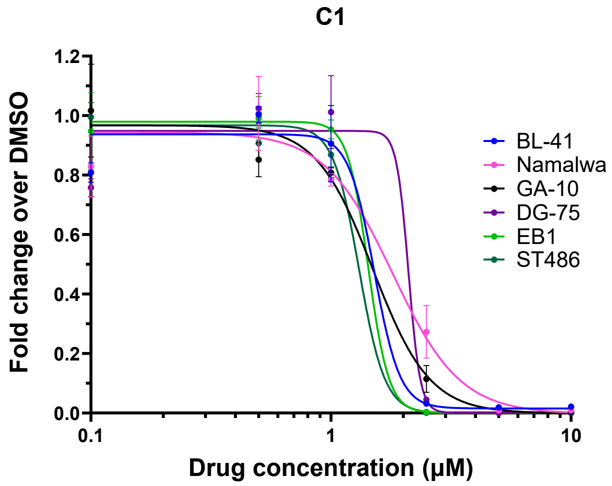

Cell line	CC_{50}
BL-41	1.51 µM
Namalwa	1.80 µM
GA-10	1.49 µM
DG-75	2.10 µM
EB1	1.61 µM
ST486	1.31 µM

Supplementary Figure S2. The dose-dependent effect of RK-33 **(A)** and C1 **(B)** on cell viability was assessed in various BL cell lines by XTT assay following 4 days of treatment with increasing concentrations of drugs. The calculated CC_{50} values for each cell line are indicated in the tables next to the curves.

Figure S3

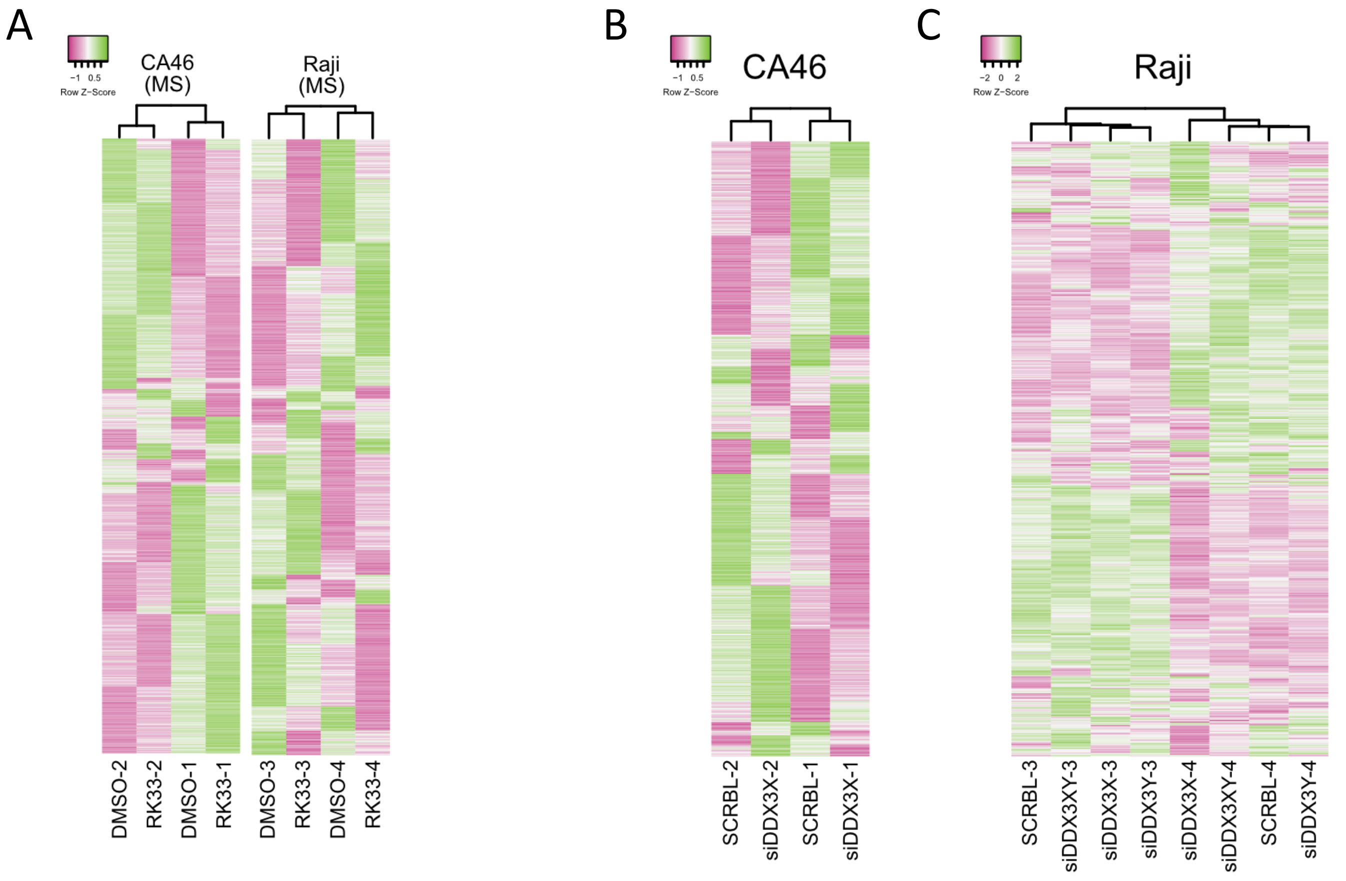

Supplementary Figure S3. Hierarchical clustering heatmaps of proteomic data that were not normalized for batch effect. **(A)** in CA46 (1137 peptides, average abundance > 50) and Raji (1010 peptides, average abundance > 50) following RK-33 or DMSO treatment. Samples were grouped into tandem mass tag (TMT) sets as follows: CA46 is TMT-1 (DMSO-1 and RK33-1) and TMT-2 (DMSO-2 and RK33-2); Raji is TMT-3 (DMSO-3 and RK33-3) and TMT-4 (DMSO-4 and RK33-4)]. (**B**) in CA46 treated with siRNA targeting DDX3X (siDDX3X) or scrambled control (SCRBL) (n = 2 per group) (961 peptides, average abundance > 50). **(C)** in Raji treated with siRNA targeting DDX3X (siDDX3X), DDX3Y (siDDX3Y), both (siDDX3XY), or scrambled control (SCRBL) (n = 2 per group) (887 peptides, average abundance > 50). In all cases, the clustering dendrograms indicates strong batch effects that mask the treatment effects.

Figure S4

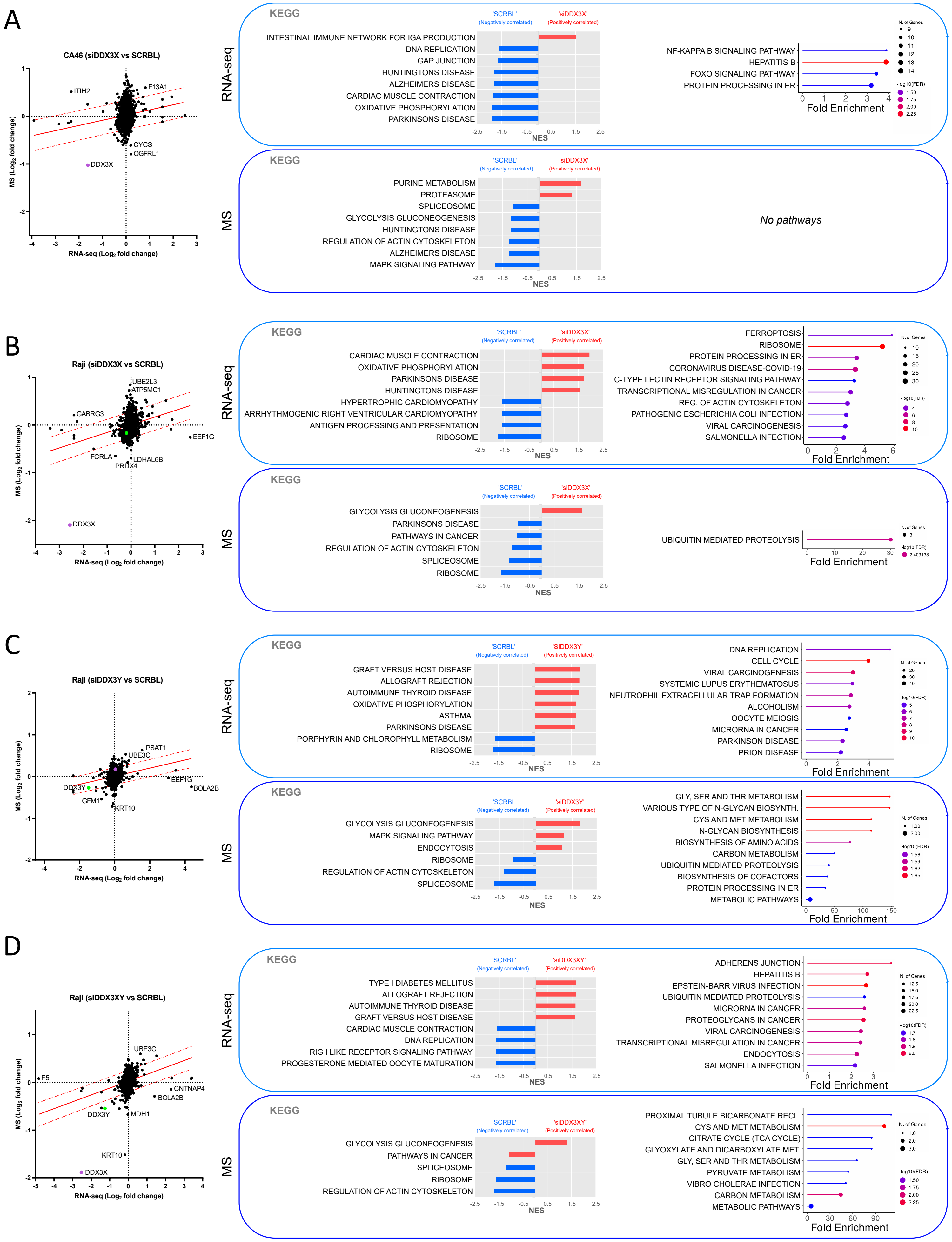

A
CA46 (siDDX3X vs SCRBL)
ITIH2
F13A1
CYCS
OGFRL1
DDX3X
MS (Log2 fold change)
RNA-seq (Log2 fold change)
RNA-seq
KEGG
'SCRBL' (Negatively correlated)
'siDDX3X' (Positively correlated)
INTESTINAL IMMUNE NETWORK FOR IGA PRODUCTION
DNA REPLICATION
GAP JUNCTION
HUNTINGTONS DISEASE
ALZHEIMERS DISEASE
CARDIAC MUSCLE CONTRACTION
OXIDATIVE PHOSPHORYLATION
PARKINSONS DISEASE
NES
NF-KAPPA B SIGNALING PATHWAY
HEPATITIS B
FOXO SIGNALING PATHWAY
PROTEIN PROCESSING IN ER
Fold Enrichment
N. of Genes
-log10(FDR)
MS
KEGG
PURINE METABOLISM
PROTEASOME
SPLICEOSOME
GLYCOLYSIS GLUCONEOGENESIS
HUNTINGTONS DISEASE
REGULATION OF ACTIN CYTOSKELETON
ALZHEIMERS DISEASE
MAPK SIGNALING PATHWAY
No pathways
B
Raji (siDDX3X vs SCRBL)
UBE2L3
ATP5MC1
GABRG3
EEF1G
FCRLA
LDHAL6B
PRDX4
DDX3X
CARDIAC MUSCLE CONTRACTION
OXIDATIVE PHOSPHORYLATION
PARKINSONS DISEASE
HUNTINGTONS DISEASE
HYPERTROPHIC CARDIOMYOPATHY
ARRHYTHMOGENIC RIGHT VENTRICULAR CARDIOMYOPATHY
ANTIGEN PROCESSING AND PRESENTATION
RIBOSOME
FERROPTOSIS
RIBOSOME
PROTEIN PROCESSING IN ER
CORONAVIRUS DISEASE-COVID-19
C-TYPE LECTIN RECEPTOR SIGNALING PATHWAY
TRANSCRIPTIONAL MISREGULATION IN CANCER
REG. OF ACTIN CYTOSKELETON
PATHOGENIC ESCHERICHIA COLI INFECTION
VIRAL CARCINOGENESIS
SALMONELLA INFECTION
GLYCOLYSIS GLUCONEOGENESIS
PARKINSONS DISEASE
PATHWAYS IN CANCER
REGULATION OF ACTIN CYTOSKELETON
SPLICEOSOME
RIBOSOME
UBIQUITIN MEDIATED PROTEOLYSIS
2.403138
C
Raji (siDDX3Y vs SCRBL)
PSAT1
UBE3C
DDX3Y
EEF1G
BOLA2B
GFM1
KRT10
'SIDDX3Y'
GRAFT VERSUS HOST DISEASE
ALLOGRAFT REJECTION
AUTOIMMUNE THYROID DISEASE
OXIDATIVE PHOSPHORYLATION
ASTHMA
PARKINSONS DISEASE
PORPHYRIN AND CHLOROPHYLL METABOLISM
RIBOSOME
DNA REPLICATION
CELL CYCLE
VIRAL CARCINOGENESIS
SYSTEMIC LUPUS ERYTHEMATOSUS
NEUTROPHIL EXTRACELLULAR TRAP FORMATION
ALCOHOLISM
OOCYTE MEIOSIS
MICRORNA IN CANCER
PARKINSON DISEASE
PRION DISEASE
'siDDX3Y'
GLYCOLYSIS GLUCONEOGENESIS
MAPK SIGNALING PATHWAY
ENDOCYTOSIS
RIBOSOME
REGULATION OF ACTIN CYTOSKELETON
SPLICEOSOME
GLY, SER AND THR METABOLISM
VARIOUS TYPE OF N-GLYCAN BIOSYNTH.
CYS AND MET METABOLISM
N-GLYCAN BIOSYNTHESIS
BIOSYNTHESIS OF AMINO ACIDS
CARBON METABOLISM
UBIQUITIN MEDIATED PROTEOLYSIS
BIOSYNTHESIS OF COFACTORS
PROTEIN PROCESSING IN ER
METABOLIC PATHWAYS
D
Raji (siDDX3XY vs SCRBL)
F5
UBE3C
CNTNAP4
BOLA2B
DDX3Y
MDH1
KRT10
DDX3X
'siDDX3XY'
TYPE I DIABETES MELLITUS
ALLOGRAFT REJECTION
AUTOIMMUNE THYROID DISEASE
GRAFT VERSUS HOST DISEASE
CARDIAC MUSCLE CONTRACTION
DNA REPLICATION
RIG I LIKE RECEPTOR SIGNALING PATHWAY
PROGESTERONE MEDIATED OOCYTE MATURATION
ADHERENS JUNCTION
HEPATITIS B
EPSTEIN-BARR VIRUS INFECTION
UBIQUITIN MEDIATED PROTEOLYSIS
MICRORNA IN CANCER
PROTEOGLYCANS IN CANCER
VIRAL CARCINOGENESIS
TRANSCRIPTIONAL MISREGULATION IN CANCER
ENDOCYTOSIS
SALMONELLA INFECTION
GLYCOLYSIS GLUCONEOGENESIS
PATHWAYS IN CANCER
SPLICEOSOME
RIBOSOME
REGULATION OF ACTIN CYTOSKELETON
PROXIMAL TUBULE BICARBONATE RECL.
CYS AND MET METABOLISM
CITRATE CYCLE (TCA CYCLE)
GLYOXYLATE AND DICARBOXYLATE MET.
GLY, SER AND THR METABOLISM
PYRUVATE METABOLISM
VIBRO CHOLERAE INFECTION
CARBON METABOLISM
METABOLIC PATHWAYS

Supplementary Figure S4. Correlation plots of RNA expression and protein abundance changes following siRNA-mediated knockdown of DDX3X (shown in purple in the plots) and DDX3Y (shown in green in the plots) in CA46 and Raji cells. **(A)** CA46 cells treated with siRNA targeting DDX3X (siDDX3X) or scrambled control (SCRBL). **(B)** Raji cells treated with siRNA targeting DDX3X (siDDX3X) or scrambled control (SCRBL). **(C)** Raji cells treated with siRNA targeting DDX3Y (siDDX3Y) or scrambled control (SCRBL). **(D)** Raji cells treated with siRNA targeting both DDX3X and DDX3Y (siDDX3XY) or scrambled control (SCRBL). For all condition, KEGG gene set enrichment analysis (GSEA, left panel in each boxed section) and ShinyGO pathway analysis (right panel) are shown for RNA-seq (top boxes) and proteomic (MS) data (botom boxes).

Figure S5

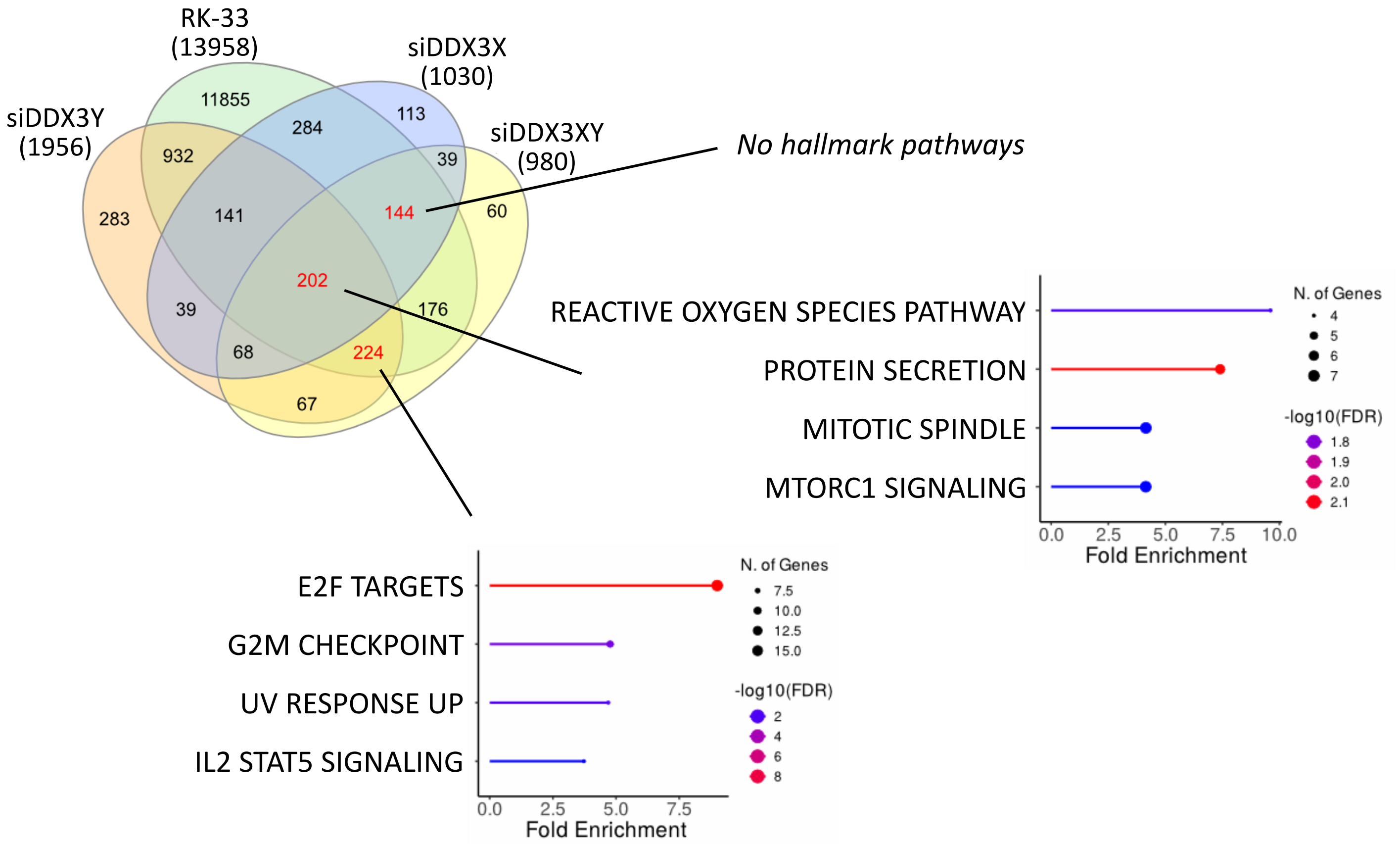

Supplementary Figure S5. Combination of the results from the inhibitor and the knockdown experiment. Hallmark pathways enriched in Raji cells following siRNA and RK-33 treatment, identified using ShinyGO (FDR<0.05). The number of differentially expressed genes per condition is indicated in the Venn diagram.

Figure S6

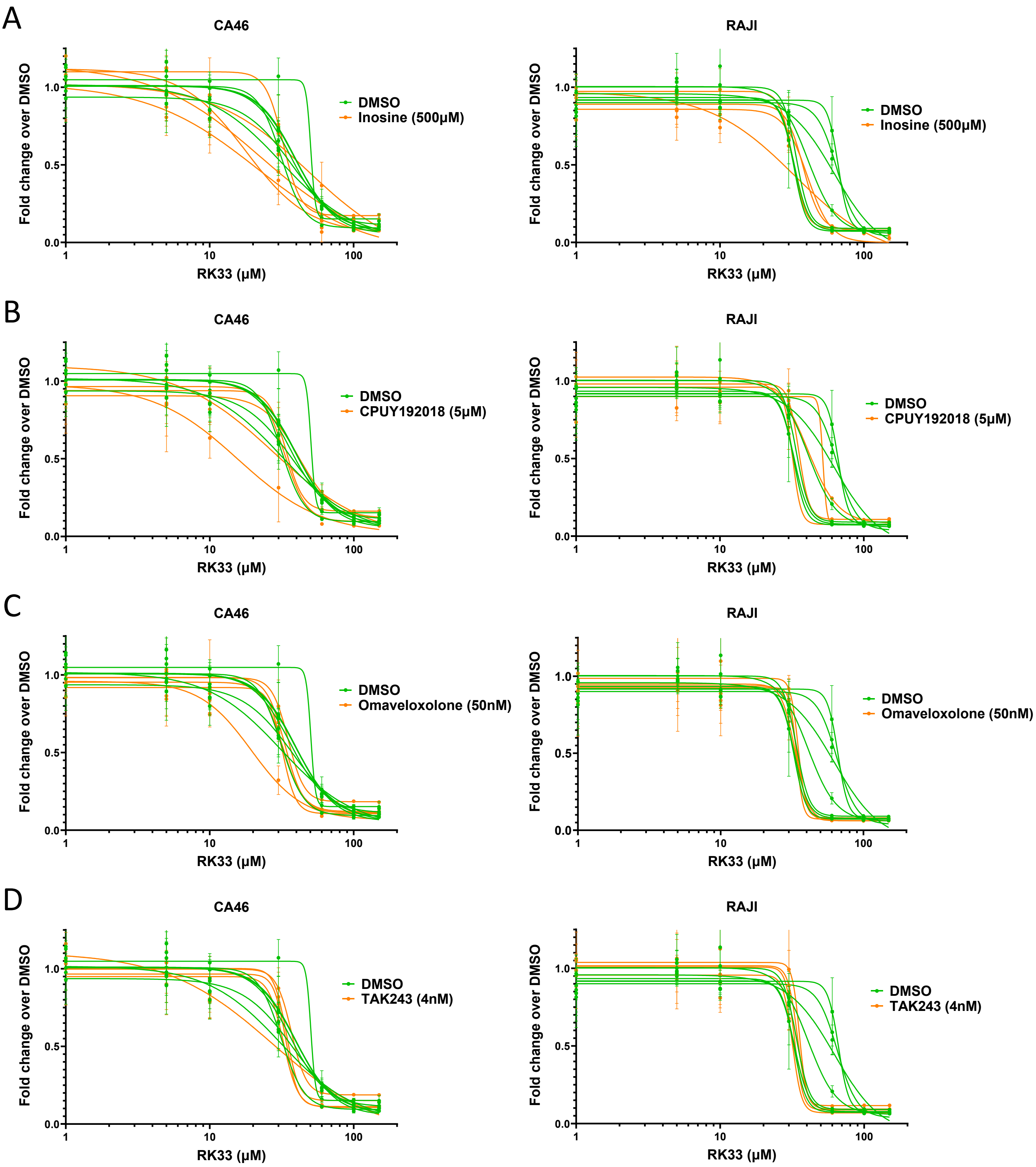

Supplementary Figure S6. Viability curves assessed by XTT viability assay after 4 days of CA46 (left) and Raji (right) cells treated with inosine **(A)**, CPUY192018 **(B)**, omaveloxolone **(C)** or TAK243 **(D)** in combination with RK-33.
